# Sugar phosphate activation of the stress sensor eIF2B

**DOI:** 10.1038/s41467-021-23836-z

**Published:** 2021-06-08

**Authors:** Qi Hao, Jin-Mi Heo, Boguslaw P. Nocek, Kevin G. Hicks, Vincent S. Stoll, Clint Remarcik, Sean Hackett, Lauren LeBon, Rinku Jain, Dan Eaton, Jared Rutter, Yao Liang Wong, Carmela Sidrauski

**Affiliations:** 1 Calico Life Sciences LLC, South San Francisco, CA USA; 2 Research & Development, AbbVie, North Chicago, IL USA; 3 Department of Biochemistry, University of Utah School of Medicine, Salt Lake City, UT USA; 4 Howard Hughes Medical Institute, University of Utah School of Medicine, Salt Lake City, UT USA; 5 Present Address: Loxo Oncology at Lilly, South San Francisco, CA USA

**Keywords:** Enzyme mechanisms, Nucleotide-binding proteins, Translation, Cryoelectron microscopy, X-ray crystallography

## Abstract

The multi-subunit translation initiation factor eIF2B is a control node for protein synthesis. eIF2B activity is canonically modulated through stress-responsive phosphorylation of its substrate eIF2. The eIF2B regulatory subcomplex is evolutionarily related to sugar-metabolizing enzymes, but the biological relevance of this relationship was unknown. To identify natural ligands that might regulate eIF2B, we conduct unbiased binding- and activity-based screens followed by structural studies. We find that sugar phosphates occupy the ancestral catalytic site in the eIF2Bα subunit, promote eIF2B holoenzyme formation and enhance enzymatic activity towards eIF2. A mutant in the eIF2Bα ligand pocket that causes Vanishing White Matter disease fails to engage and is not stimulated by sugar phosphates. These data underscore the importance of allosteric metabolite modulation for proper eIF2B function. We propose that eIF2B evolved to couple nutrient status via sugar phosphate sensing with the rate of protein synthesis, one of the most energetically costly cellular processes.

## Abstract (web summary)

The activity of translation initiation factor eIF2B is known to be modulated through stress-responsive phosphorylation of its substrate eIF2. Here, the authors uncover the regulation of eIF2B by the binding of sugar phosphates, suggesting a link between nutrient status and the rate of protein synthesis.

## Introduction

Eukaryotic translation initiation factor 2B (eIF2B) is the decameric guanine nucleotide exchange factor (GEF) for the GTPase eukaryotic translation initiation factor 2 (eIF2). The essential function of these two complexes in cells is the delivery of the initiating methionine to the ribosome to allow protein synthesis. Upon delivery of the initiator Met-tRNA_i_ to the site of translation initiation, bound GTP is hydrolyzed and eIF2 is released from the scanning ribosome. eIF2B catalyzes GDP-GTP exchange on eIF2, enabling rebinding of Met-tRNA_i_ to allow for a new cycle of mRNA translation initiation^1^.

Because of the energetic and biosynthetic costs of protein synthesis, translation initiation is a highly regulated process. Four kinases respond to diverse stresses by phosphorylating the α subunit of eIF2, converting it from a substrate into a tight-binding competitive inhibitor of eIF2B^2,3^. The resulting decrease in formation of the active eIF2-GTP-Met-tRNA_i_ ternary complex reduces translation initiation and triggers the Integrated Stress Response (ISR). Its activation attenuates bulk protein synthesis while stimulating translation of a subset of transcripts, such as the transcription factor ATF4, that promote adaptation under stress conditions^4–6^. Both of these ISR effects are important for cells to adapt to acute stress conditions but may lead to dysfunction when chronically engaged.

eIF2B is an enzyme of considerable complexity, comprising five different subunits arranged in a two-fold symmetric structure to form a decamer^7–10^. Two catalytic subcomplexes, each comprised of a γ and ε subunit, decorate the central regulatory core, which is a heterohexamer composed of two α, β and δ subunits. We and others demonstrated that small molecule eIF2B activators bind at the axis of symmetry, acting as molecular staplers that stabilize and activate the decameric holoenzyme to attenuate the ISR^9–13^. Furthermore, we showed that partial loss-of-function mutations in eIF2B that cause Vanishing White Matter (VWM), a devastating neurodegenerative disease, destabilize the eIF2B decamer and reduce its GEF activity. Stabilization of the decamer with eIF2B activators rescued the GEF activity of these mutations both in vitro and in vivo, revealing the importance of the decameric complex for normal eIF2B function^13^.

Curiously, the eIF2B regulatory core is related to an ancient family of homo-hexameric sugar phosphate-metabolizing enzymes, with conservation observed between eIF2Bα/β/δ and the archaeal ribose-1,5-bisphosphate isomerase (RBPI)^14–16^. Why was a metabolic enzyme co-opted to serve as the core for a multi-subunit GEF? One enticing hypothesis is that this might enable direct coupling of nutrient availability to protein synthesis, the most energy-intensive process in the cell. Sugar phosphorylation is the first step in the catabolism of carbohydrates to drive ATP generation and biomass accumulation. The eIF2α kinase GCN2 senses amino acid availability, whereas the other kinases evolved to detect non-nutrient stressors^17,18^. None of these kinases sense carbon or ATP abundance, and phosphorylation itself is an energy-consuming act, thus an ancient and direct role for eIF2B in energy-sensing is compelling.

We note that the origins of our hypothesis are over 30 years old. Gross et al. proposed that eIF2B activity could be matched to energy availability, based on observations that glucose-6-phosphate was required to maintain mRNA translation rates in cell lysates^19^. Contemporaneously, Wahba et al. suggested that pyridine dinucleotides (NAD^+^/NADP^+^/NADPH) served a role in controlling eIF2 ternary complex turnover^20^. These early works were hampered by a lack of readily available metabolites to test, and an inability to use fully reconstituted in vitro systems. Eventually, the community directed its attention to studying the regulation of eIF2B by phosphorylated eIF2α. More recently, Kuhle et al. used isothermal titration calorimetry (ITC) to demonstrate binding of the monophosphate ribonucleotides AMP and GMP to recombinant *C. thermophilum* eIF2Bα^16^. This prompted us to revisit the concept of nutrient status as a regulator of the eIF2B-eIF2 axis, with a focus on direct sensing by eIF2B.

In this work, we conduct two orthogonal unbiased screens for potential metabolite ligands of eIF2B, identifying sugar phosphates as eIF2Bα binders and enzymatic activators. Using cryo-EM and X-ray crystallography, we elucidate structures of sugar phosphates bound to eIF2B and demonstrate that they occupy the ancestral catalytic pocket within the eIF2Bα subunit. We further show that sugar phosphates stabilize the decameric holoenzyme and enhance its GEF activity similar to synthetic eIF2B activators. However, these natural metabolites exert their effect by engaging the eIF2Bα_2_ dimer, rather than by bridging the eIF2B(β/δ)_2_ tetramer interface like ISRIB. Unlike the recently demonstrated activity of ISRIB as an antagonist of phosphorylated eIF2 binding^21,22^, the sugar phosphate mechanism is primarily driven by its ability to decamerize eIF2B. We generate point mutations in the eIF2Bα sugar-binding pocket, one of which is known to cause VWM disease. These mutations abolish metabolite binding and the concomitant increases in decamer stabilization and activity, raising the possibility that impaired sugar phosphate sensing by eIF2B may contribute to disease etiology. In sum, our results underscore the importance of sugar phosphate metabolites in modulation of eIF2B activity.

## Results

### An unbiased screening approach identifies sugar phosphates as ligands of eIF2Bα

Beginning with our hypothesis that eIF2B may be a direct nutrient sensor, we reasoned that the eIF2Bα/β/δ regulatory subunits could be ideal candidates for an unbiased metabolite screen by virtue of their homology to aforementioned sugar-metabolizing enzymes. To this end, we employed mass spectrometry integrated with equilibrium dialysis for the discovery of allostery systematically (MIDAS)^23^. Due to the high protein concentrations required for the MIDAS approach, we focused our efforts on the small 34 kDa eIF2Bα subunit, which can be purified in large quantities and forms a stable homodimer (eIF2Bα_2_) in solution. By contrast, eIF2Bβ/δ form a 227 kDa heterotetrameric subcomplex with the γ and ε subunits^8^.

**Fig. 1:**
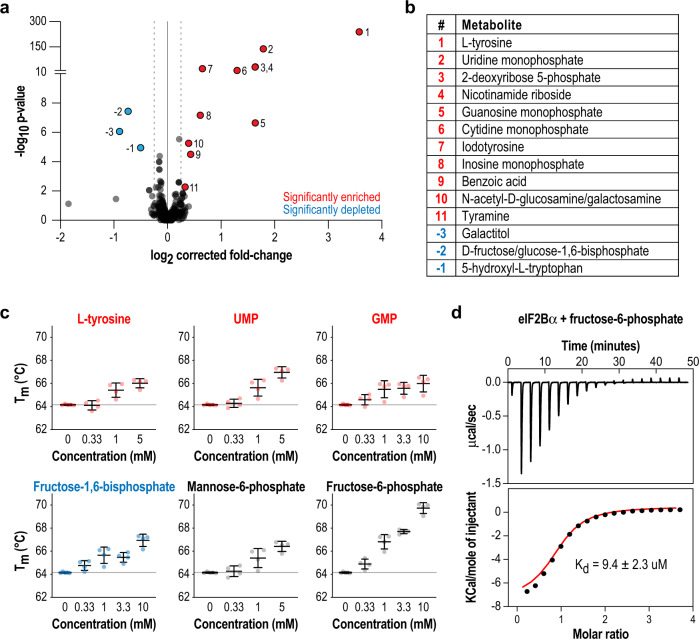
Unbiased screening by MIDAS identifies putative ligands that interact with eIF2Bα. **a** Volcano plot of metabolites analyzed in MIDAS, comparing the fold-change between the protein chamber and metabolite chamber. Red indicates metabolites that were significantly enriched in the protein-containing chamber, whereas blue indicates metabolites that were significantly depleted (q < 0.1) based on a two-tailed Wald test. The full data are available as Supplementary Data 1. **b** All 16 significant hits from the MIDAS binding screen numbered in **a** are tabulated. **c** Differential scanning fluorimetry of eIF2Bα in combination with selected metabolites in dose–response. Metabolite binding increased the T_m_ of eIF2Bα. Bars are mean ± standard deviation of *n* = 4 independent experiments. Color coding in **b**, **c** are as in **a**. **d** K_d_ of the eIF2Bα–F6P interaction measured by ITC. The upper subpanel shows the baseline-subtracted thermogram. The bottom subpanel represents the binding isotherm, with the red line indicating the fit curve.

MIDAS screening of 412 naturally occurring human metabolites against eIF2Bα_2_ yielded 16 that were enriched or depleted using cutoffs of log_2_(fold-change) < −0.25 or > 0.25, q < 0.1 (Fig. 1a, b, Supplementary Data 1). Enriched hits are metabolites that are more concentrated in the eIF2Bα-containing chamber, consistent with direct physical interaction. Depleted hits in MIDAS are more complicated to interpret and can indicate tight physical interactors, covalent modifications of metabolites to the target protein, or enzymatic substrates that are consumed in the protein chamber at a rate faster than the metabolite diffusion rate across the chambers, leading to an apparent loss of the metabolite. The amino acid tyrosine was the most enriched metabolite identified, and its derivative iodotyrosine was also significantly enriched. Interestingly, 11/16 of the hits were sugars, of which 8 were sugar phosphates (including ribonucleotides) similar to the substrate of RBPI, ribose-1,5-bisphosphate.

To orthogonally validate the MIDAS hits, we performed differential scanning fluorimetry on a subset of metabolites to assess their effect on the thermal stability of eIF2Bα_2_ (Fig. 1c, Supplementary Fig. 1a). We also assayed a number of sugar phosphates that did not reach statistical significance in MIDAS, recognizing that they may not have met the significance threshold due to the inability of our mass spectrometry method to distinguish between identical sugar masses (glucose, mannose, galactose, and fructose), resulting in signal splitting across 7 different sugar phosphate isomers. The MIDAS hits increased the melting temperature (T_m_) of eIF2Bα_2_ in a concentration-dependent manner. Remarkably, fructose-6-phosphate (F6P), which was not a MIDAS hit, induced the largest T_m_ shift of 6 °C (Fig. 1c).

Given the marked increase in thermal stability induced by F6P and its key position in central carbon metabolism, we examined its interaction with eIF2Bα in more detail. ITC measurements showed that it binds to eIF2Bα with K_d_ = 9.4 ± 2.3 μM (Fig. 1d). By contrast, the affinity of eIF2Bα for the F6P isomer mannose-6-phosphate (M6P) was considerably weaker, with K_d_ = 282 ± 35 μM (Supplementary Fig. 1b). Although there is considerable uncertainty about free intracellular metabolite concentrations (and in vivo values are likely to be highly dependent on cell type, nutrient status, and other factors), these values are consistent with reported values of many sugar phosphates in the 1–100 μM range^24^. In summary, the unbiased screen and ensuing characterization indicated that sugar phosphates are ligands for eIF2Bα at physiologically relevant concentrations.

### Unbiased activity-based screening identifies 5′/6′ sugar phosphates as eIF2B activators

**Fig. 2:**
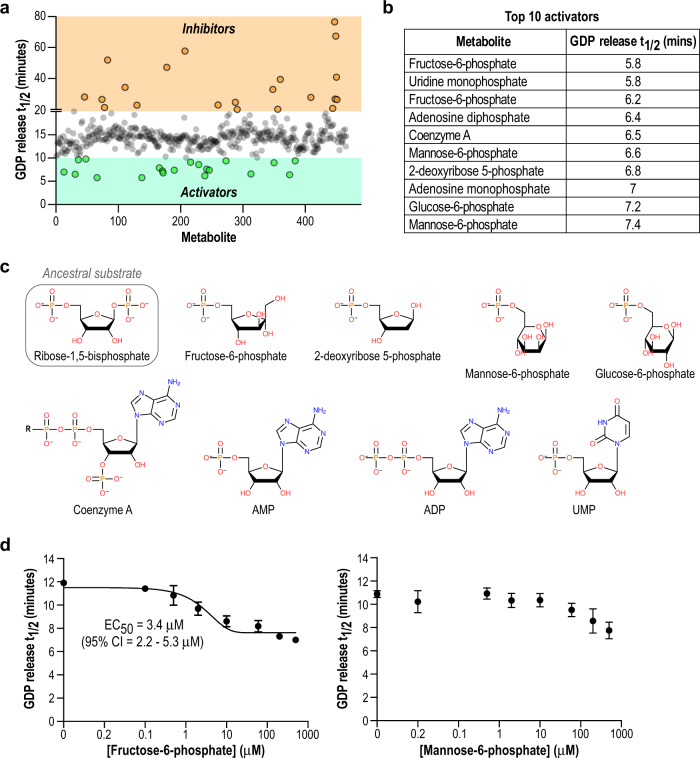
Unbiased activity-based screening identifies 5′/6′ sugar phosphates as eIF2B activators. **a** Plot of individual metabolites arrayed against GDP release t_1/2_ derived from the GEF activity assay. Based on the basal activity of eIF2B under these assay conditions, a t_1/2_ cutoff of <10 min was used to define activators (green) and a cutoff of >20 min was used to define inhibitors (orange). **b** List of the top 10 activators from the screen, ordered by GDP release t_1/2_. The full data are available as Supplementary Data 2. **c** Structures of the top 10 activators from the screen, compared to the substrate of archaeal RBPI. The sugar ring and 5′/6′ phosphate moiety are common structural motifs. **d** GDP release t_1/2_ of eIF2B using increasing concentration of F6P or M6P. Only the eIF2B + F6P data could be fit to a dose-response curve with *R*_2_ > 0.9. Each point represents mean ± standard deviation of 3 technical replicates.

The primary function of eIF2B is to catalyze the exchange of GDP for GTP on its cognate substrate eIF2. Our results thus far were highly suggestive of a bona fide interaction between sugar phosphates and eIF2Bα, but MIDAS does not report on the effect of binding on eIF2B function. We and others previously described fluorescence-based assays to monitor eIF2B GEF activity on eIF2^10,12,25^. Hence, we used an arrayed version of the MIDAS metabolite library to conduct an orthogonal unbiased screen based on modulation of eIF2B activity (Fig. 2a, Supplementary Data 2).

At a screening concentration of 10 μM, the top 10 activators of eIF2B GEF activity were all sugars with a 5′ or 6′ phosphate moiety, consistent with the structure of the ancestral substrate of this complex (Fig. 2b, c). Some metabolites, such as F6P and M6P, were represented twice in the physical screening library, and the activity screen positioned both replicates of each metabolite within the most activating hits, increasing our confidence of their significance. UMP, a strong hit in MIDAS, was also a hit in this functional assay screen, together with other ribonucleotides such as AMP and ADP. We attribute their effect to the ribophosphate portion of the molecule, rather than the base group (see structure data below). Tyrosine, which was a prominent ligand identified by MIDAS, did not show an appreciable effect on eIF2B GEF activity in vitro, thus we did not pursue it further. Whether it has a regulatory role in complex assembly in cells remains to be determined.

We further investigated the stimulatory effects of F6P and M6P on GEF activity by performing dose–response experiments with eIF2B (Fig. 2d). As suggested by the thermal shift and ITC results (Fig. 1c, d, Supplementary Fig. 1b), F6P is a more potent activator (EC_50_ = 3.4 μM) of eIF2B activity than M6P. Our activity screen also identified a set of inhibitors, including bile salts and a variety of acidic compounds (Fig. 2a, Supplementary Data 2). None of the identified inhibitors appeared to interact with eIF2Bα based on MIDAS, but we cannot rule out potential interactions with the eIF2B holoenzyme. Nevertheless, there was no obvious structural commonality between inhibitors, likely because there are many possible ways to non-specifically interfere with an enzymatic assay, but far fewer ways to enhance activity. Thus, we constrained our further investigation to understanding the mechanism of the sugar phosphates as activators.

### Sugar phosphates bind to the evolutionarily conserved substrate binding pocket in eIF2Bα

**Fig. 3:**
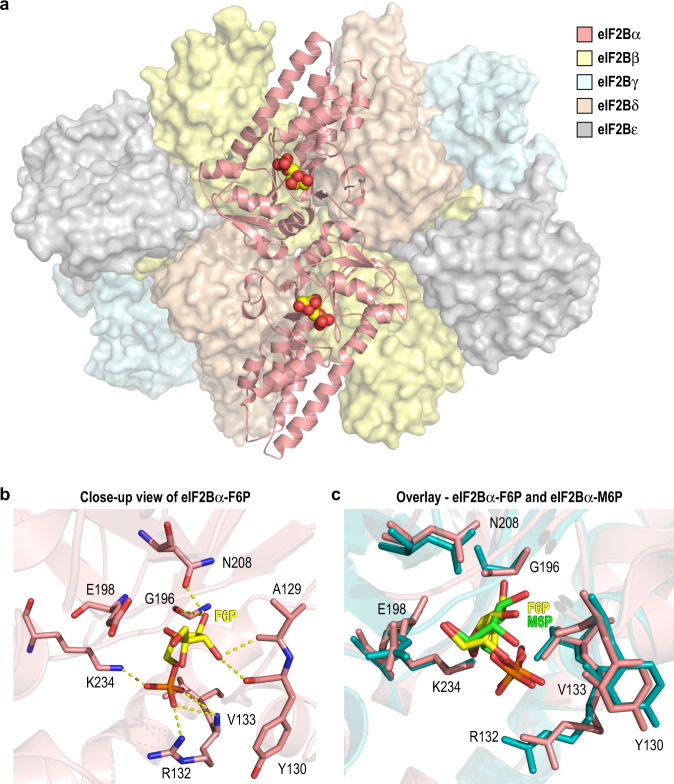
2.9 Å cryo-EM structure of eIF2B in complex with F6P. **a** Overall structure of the eIF2B-F6P complex with eIF2Bα in cartoon representation and eIF2Bβ/δ/γ/ε represented as surfaces (PDB 7KMF). The single F6P molecule bound within each eIF2Bα monomer is shown as space-filling spheres. **b** Close-up view of the eIF2Bα metabolite binding pocket, with residues contributing to the F6P interaction shown in stick representation. H-bonds are represented by dashed yellow lines. **c** Overlay of the sugar phosphate binding pockets in the eIF2B-F6P cryo-EM structure (pink) and the eIF2Bα-M6P crystal structure (PDB 7KMA; teal). F6P and M6P are shown as yellow and green sticks, respectively.

Motivated by our biochemical evidence that sugar phosphates are activators of eIF2B, we determined a 2.9 Å cryo-EM structure of eIF2B bound to F6P, the highest affinity identified ligand (Supplementary Figs. 2 and 3). The eIF2B-F6P complex shares the same overall architecture as previously reported eIF2B structures (Fig. 3a)^9,10^. F6P occupies an inter-domain cavity within each eIF2Bα monomer (chains G and H), which is the conserved metabolite binding pocket in the ancestral eIF2Bα/β/δ homologs^16,26^. This pocket is on the opposite face of the eIF2B holoenzyme from the previously described ISRIB binding pocket that sits at the symmetric eIF2Bβ/δ interface. Protein-ligand interactions in the F6P pocket are mediated by both the sugar and phosphate moieties. G196, A129, Y130, E198, and N208 form H-bonds with the F6P pyranose ring, while the positively charged residues R132 and K234 coordinate the phosphate group (Fig. 3b). The 1′ position of F6P is exposed to solvent, suggesting a way by which bulkier ribonucleotides (e.g. UMP and AMP) could be accommodated in this site.

Notably, we did not observe electron density for any ligands within the eIF2Bβ or eIF2Bδ metabolite pockets, despite their homology to eIF2Bα. An examination of their amino acid sequences revealed that key residues involved in sugar ring coordination (E198 and N208) are conserved between eIF2Bα and two homologs from the same enzyme family with known metabolite binding activity, but not conserved in eIF2Bβ/δ (Supplementary Fig. 4a). This pattern is maintained in yeast eIF2B subunit sequences, suggesting that the functional divergence of the eIF2Bα/β/δ paralogs is ancient and may have co-evolved with the introduction of eIF2 phosphorylation as a mode of regulation. Consistent with our observation, ITC measurements showed that F6P did not interact appreciably with an eIF2B(βδγε) subcomplex (Supplementary Fig. 4b). An eIF2Bα homolog, human methylthioribose-1-phosphate isomerase (MTNA), binds to a sugar with a 1′ phosphate group. While residues in MTNA participating in sugar coordination are conserved with eIF2Bα, residues involved in phosphate coordination have diverged, likely due to the difference in phosphate group position (Supplementary Fig. 4a). We confirmed the importance of the phosphate position by demonstrating that eIF2Bα did not interact with the F6P isomer glucose-1-phosphate (G1P; Supplementary Fig. 4c).

To confirm that the binding mode for F6P is shared with other sugar phosphates, we determined the 2.7 Å crystal structure of eIF2Bα bound to M6P (Supplementary Figs. 5 and 6). Comparison of the F6P and M6P structures revealed a metabolite recognition mechanism employing the same residues in the eIF2Bα pocket (Fig. 3c). Slight differences in bonding due to the larger size of the M6P sugar could account for the difference in affinity between F6P and M6P.

To identify potential changes in protein conformation upon sugar phosphate binding, we performed pairwise comparisons of eIF2B-F6P and eIF2Bα-M6P with the reported structures of apo eIF2B (PDB 7D46^22^) and eIF2Bα (PDB 3ECS^27^). eIF2Bα-M6P aligned to apo eIF2Bα with r.m.s.d. = 0.44 Å, indicating high overall similarity (Supplementary Fig. 7a). Curiously, the metabolite binding site in the apo structure is occupied by SO_4_ molecules from the crystallization buffer. This could suggest that the site must be filled for optimal protein stability. The free eIF2Bα structures also align with eIF2Bα in the context of the holoenzyme (with or without F6P) with r.m.s.d. < 0.9 Å, and interdomain orientations are not significantly changed (Supplementary Fig. 7b).

The holoenzyme structures in the absence and presence of F6P are also strikingly similar, with global r.m.s.d. = 1.6 Å. The most prominent difference is a uniform shift in the position of both eIF2Bε subunits, wherein the β-sheet rich domain moves inwards towards the cognate eIF2Bα subunit by ~1.5–2 Å (Supplementary Fig. 7c). This results in a slightly compacted conformation of the holoenzyme in the presence of F6P, although all major structural features are otherwise preserved. Together, our data indicate that sugar phosphates interact with eIF2B exclusively through the α subunit, and that conserved residues in eIF2Bα confer selective binding via both the sugar and phosphate moieties.

### Sugar phosphate binding by the α subunit enhances eIF2B decamer formation

We previously showed that eIF2B GEF activity is correlated with the stability of the decameric holoenzyme^12^. Indeed, the small molecule eIF2B activator ISRIB functions by serving as a molecular stapler between two eIF2B(βδγε) subcomplexes^9,10^. In nature, the eIF2Bα_2_ dimer has an analogous function as it promotes dimerization of these subcomplexes by bridging the two-fold symmetric interface of the holoenzyme. We hypothesized that sugar phosphate binding might enhance the ability of eIF2Bα_2_ to promote this dimerization event and thus activation of the complex.

**Fig. 4:**
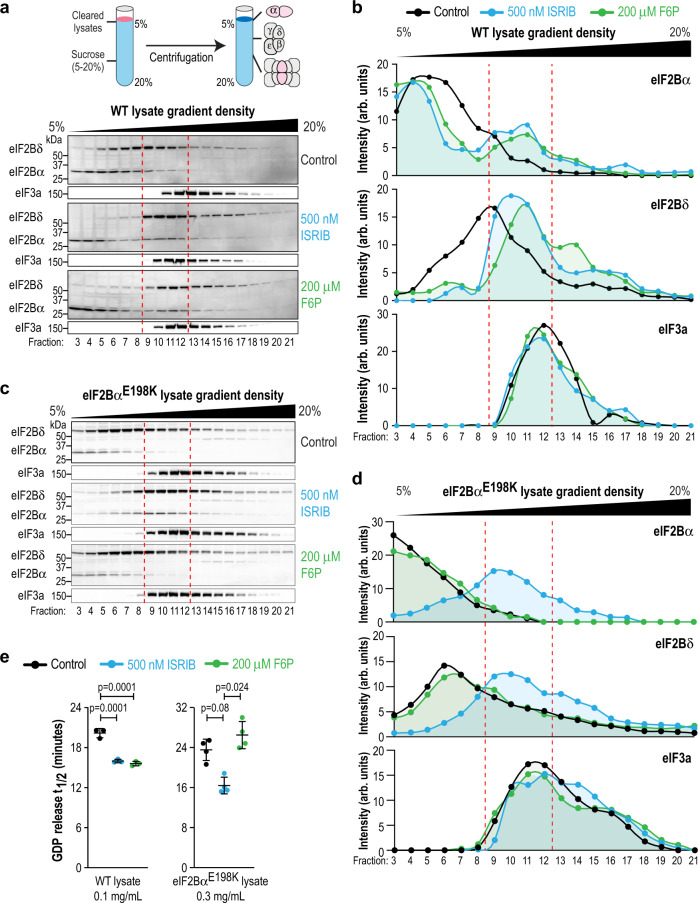
Sugar phosphate binding by the α subunit enhances eIF2B decamer formation. **a**–**d** eIF2B complex assembly from WT and eIF2Bα^E198K^ HEK293T lysates treated with ISRIB (blue) or F6P (green) was monitored by sucrose gradient centrifugation. Fractions from the sucrose gradient were subjected to SDS-PAGE followed by immunoblotting with the indicated antibodies. eIF3a was used as an internal control. Western blot data in **a**, **c** are quantified in **b**, **d**, respectively. Data shown are representative of 2–3 replicates of each experiment. Bands were normalized by the total intensity of each subunit in its respective gradient. Dashed red lines demark the boundary of the decameric eIF2B peak. WT eIF2B forms a decamer in the presence of both ISRIB and F6P. By contrast, eIF2Bα^E198K^ complexes respond to ISRIB but not F6P. **e** GDP release t_1/2_ in a GEF assay using lysates from WT or eIF2Bα^E198K^ cells. WT lysate activity is stimulated by both ISRIB and F6P, whereas eIF2Bα^E198K^ lysate does not respond to F6P. Bars are mean ± standard deviation of *n* = 3 independent experiments of 3 technical replicates each. Statistical significance was tested by one-way ANOVA with Tukey’s multiple testing correction.

To examine this possibility, we chose to directly assess the effect of F6P binding on eIF2B decamer formation. We generated lysate from wild-type (WT) HEK293T cells and subjected them to sucrose gradient centrifugation in the presence of different ligands to separate the various assembly states of eIF2B (α_2_, βδγε and [αβδγε]_2_). In this assay, we used a supraphysiological salt concentration (400 mM KCl) to increase our sensitivity to detect transitions between assembly states. Subcomplexes were tracked and quantified using antibodies directed against eIF2Bα and eIF2Bδ. As previously reported, ISRIB caused eIF2Bα and eIF2Bδ to co-migrate into a denser portion of the gradient, indicating formation of the decameric holoenzyme (Fig. 4a, b, fractions 9–12, demarked by the red dashed lines)^11^. Remarkably, F6P induced stabilization of the eIF2B decamer comparable to ISRIB. We confirmed that the identity of the sugar phosphate is important by showing that G1P, an inactive metabolite in the enzymatic assay, did not affect the migration of eIF2B subunits (Supplementary Fig. 8a–c). Migration of eIF3a, which is a constituent of another high molecular weight translation initiation complex, was not affected by any of the treatments, confirming the specificity of ISRIB and F6P.

As an orthogonal approach to assess complex formation, we performed size-exclusion chromatography on recombinant eIF2B subunits in the presence and absence of ISRIB or F6P (Supplementary Fig. 9a, b). As expected, ISRIB enhanced formation of an (βδγε)_2_ octamer in the absence of eIF2Bα, and the (αβδγε)_2_ decamer in the presence of eIF2Bα. By contrast, and consistent with its exclusive binding to eIF2Bα, F6P was ineffective at promoting complex formation in the absence of eIF2Bα.

Based on structural analysis of the binding pocket, we generated a HEK293T cell line bearing a homozygous knock-in mutation of eIF2Bα^E198K^, which we predicted would disrupt coordination of a ligand’s sugar moiety. We validated our prediction by demonstrating that purified, recombinant eIF2Bα^E198K^ exhibited 11-fold reduced affinity for F6P in an ITC assay (Supplementary Fig. 8d; compare with Fig. 1d). We attempted to generate a second knock-in cell line with another binding-disrupting mutant, eIF2Bα^N208Y^, but were unsuccessful (see next section and Discussion). We subjected eIF2Bα^E198K^ cell lysate to the same sucrose gradient analysis as above. As expected, ISRIB produced a shift to the decameric form in the mutant lysate, as it binds to the eIF2B(βδγε) subcomplex (Fig. 4c, d). However, eIF2Bα^E198K^ complexes were unresponsive to F6P, confirming our hypothesis that sugar phosphate binding results in eIF2Bα-mediated holoenzyme formation.

Finally, we strengthened the mechanistic link between decamer formation and enzymatic activity by showing that the GEF activity of eIF2Bα^E198K^ lysate was boosted in the presence of ISRIB but not F6P (Fig. 4e). In these assays, we used a greater amount of mutant lysate than WT to achieve comparable enzymatic activity, as the mutation reduced the levels of eIF2B subunits, a phenomenon we have previously reported (Supplementary Fig. 8e, see Discussion)^12^. Interestingly, in WT eIF2B, we observed a similar boost in GEF activity using either ISRIB or F6P, but no additive effects when the two ligands were combined (Supplementary Fig. 9c). This is consistent with our hypothesis that these ligands converge upon a common pathway, i.e., eIF2B decamerization, to exert their activating effects.

Recent work showed that in addition to promoting eIF2B decamer formation, ISRIB can also enhance the enzymatic activity of this complex by allosterically antagonizing the interaction of eIF2B with its substrate-turned-inhibitor phospho-eIF2^21,22^. Motivated by this exciting finding, we investigated whether F6P elicits a similar effect. To this end, we used HEK293T cells harboring a knock-in C-terminal FLAG tag at the endogenous eIF2Bβ locus. We treated these cells with thapsigargin, an inhibitor of the endoplasmic reticulum Ca^2+^ ATPase, to generate an intracellular pool of phospho-eIF2α. Consistent with published work, we observed co-immunoprecipitation of phospho-eIF2α with FLAG-tagged eIF2B that was significantly disrupted by ISRIB (Supplementary Fig. 10a, b)^21,22^. By contrast, F6P did not decrease the interaction between phospho-eIF2α and eIF2B under these conditions, revealing a mechanistic difference between these two eIF2B activators.

### Contrasting effects of sugar phosphate binding in two eIF2Bα VWM disease mutants

**Fig. 5:**
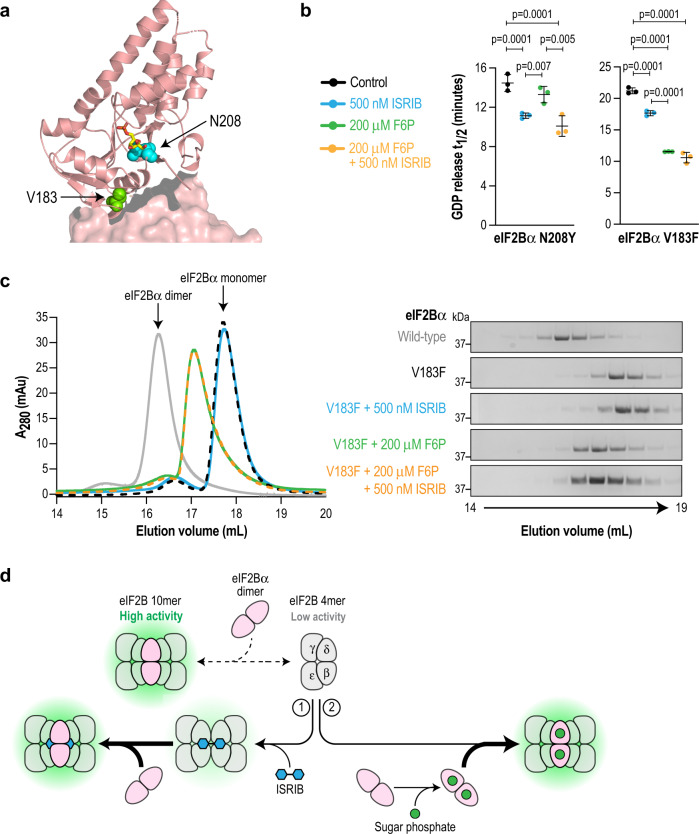
F6P enhances decamer formation and activity of the VWM mutant eIF2Bα^V183F^ but not eIF2Bα^N208Y^. **a** Close-up view of a single eIF2Bα monomer showing the positions of residues V183 (green) and N208 (cyan). F6P is shown in stick representation. N208 is within the binding pocket and V183 is positioned at the interface with another eIF2Bα subunit. **b** GDP release t_1/2_ in a GEF assay using recombinant eIF2B reconstituted with either eIF2Bα^N208Y^ or eIF2Bα^V183F^. N208Y activity is stimulated by ISRIB (blue) but not F6P (green), whereas the reverse is true for V183F. Both mutants are stimulated by the combination of ISRIB and F6P (orange). Bars are mean ± standard deviation of *n* = 3 independent experiments of 3 technical replicates each. Statistical significance was tested by one-way ANOVA with Tukey’s multiple testing correction. **c** Size-exclusion chromatography of purified recombinant wild-type or V183F eIF2Bα in the presence of ISRIB and/or F6P. UV absorbance chromatograms as well as Coomassie-stained fractions are shown. Data shown are representative of 3 independent replicates. Wild-type eIF2Bα is a dimer whereas eIF2Bα^V183F^ is a monomer, but is shifted towards a dimeric form by F6P. **d** Model depicting two distinct pathways to achieve eIF2B decamerization and activation. Arrow thickness indicates the rate of a reaction occurring. The synthetic activator ISRIB bridges the eIF2Bβ/δ interface to form an octamer, which then interacts with eIF2Bα_2_. Sugar phosphates bind to eIF2Bα_2_ and promote its interaction with eIF2B(βδγε) to form the holoenzyme.

Among the conserved residues in the eIF2Bα metabolite pocket, the sugar-coordinating N208 was of particular interest because an eIF2Bα^N208Y^ mutation has been reported in a patient with VWM disease^28^. To study the effect of this mutation, we chose to contrast it against another VWM mutation in eIF2Bα that we previously characterized, V183F, which does not reside in the binding pocket (Fig. 5a). Instead, eIF2Bα^V183F^ is a severe VWM mutation that localizes to the interface of the α_2_ homodimer and disrupts dimer formation as well as decamerization of the holoenzyme^8,12,29^.

We purified recombinant versions of both eIF2Bα VWM mutants and combined them with WT eIF2B(βδγε) to reconstitute the mutant holoenzymes. As predicted, we observed that eIF2Bα^N208Y^ abrogates binding to F6P (Supplementary Fig. 11a), whereas eIF2Bα^V183F^ retains WT affinity for this ligand (K_d_ = 6.3 ± 0.7 μM; Supplementary Fig. 11b). The basal GEF activity of eIF2B reconstituted with eIF2Bα^N208Y^ was modestly reduced compared to WT (14.5 ± 0.9 min; Fig. 5b) and this activity was responsive to ISRIB but not to F6P (11.2 ± 0.3 min for ISRIB, *p* = 0.0026 vs control; 13.3 ± 0.8 min for F6P, *p* = 0.25 vs control). As we previously reported, eIF2B reconstituted with eIF2Bα^V183F^ exhibits markedly reduced basal GEF activity that is only partially restored by ISRIB (21.2 ± 0.5 min and 17.7 ± 0.1 min, respectively; Fig. 5b). Surprisingly, incubation with F6P caused a dramatic increase in the activity of V183F mutant eIF2B (11.5 ± 0.1 min). As with WT eIF2B, combining the two ligands did not yield further enhancement of activity.

A potential explanation for the striking rescue of eIF2Bα^V183F^ activity by F6P is through stabilization of the compromised α_2_ homodimer, which then promotes decamer formation. To evaluate this possibility, we subjected eIF2Bα^V183F^ to size-exclusion chromatography and followed its fate after addition of F6P or ISRIB. Consistent with previous reports^8^, wild-type eIF2Bα migrates as a dimer whereas eIF2Bα^V183F^ migrates as a monomer (Fig. 5c, gray line and dashed black line, respectively), highlighting the crippling effect of this mutation. Notably, F6P induced a partial shift from the monomer to the dimer peak, demonstrating a stabilizing effect on the VWM mutant. As expected, ISRIB had no effect on the α subunit.

To investigate the effects of F6P and ISRIB on V183F holoenzyme formation, we subjected eIF2Bα^V183F^ mutant lysates to sucrose gradient centrifugation. ISRIB and F6P promoted decamer formation at comparable levels (Supplementary Fig. 11c, d). We confirmed this observation by using a size-exclusion chromatography assay with recombinant complexes (Supplementary Fig. 11e). However, the higher sensitivity of the latter assay enabled us to detect a significant reduction of the eIF2Bα^V183F^ monomer peak in the presence of F6P. Collectively, the data indicate that dimerization of V183F eIF2B can be elicited by promoting eIF2Bα^V183F^ dimerization, or by stapling eIF2B(βδγε) subcomplexes.

As mentioned above, we were unable to generate viable cells harboring the eIF2Bα^N208Y^ mutation. At face value, the eIF2Bα^N208Y^ data suggest that recognition of sugar phosphates may be important in maintaining eIF2Bα stability and/or regulating eIF2B function in a physiological context. Inability to respond may be a possible route that leads to VWM disease, either by loss of specific activity or destabilization of the holoenzyme. However, even robust binding to metabolites is not sufficient if other aspects of protein stability are compromised, as evinced by the eIF2Bα^V183F^ mutant.

## Discussion

eIF2B is unique among GEFs in its size and complexity, a property which has not been well understood. Although the GEF catalytic domain is located in the ε subunit, formation of the decameric complex is necessary for full activity, as it forms a scaffold for interaction with its substrate eIF2^30–33^. In previous work, we and others elucidated the mechanism by which synthetic eIF2B activators (ISRIB and 2BAct) directly staple the two halves of the eIF2B decamer together by bridging the regulatory β and δ subunits^9,10,12^. More recently, ISRIB was shown to act as an allosteric antagonist of phospho-eIF2α binding^21,22^. The evidence presented suggests that this is its primary mechanism of action in cells with high levels of eIF2B subunits, and importantly, sufficient levels of the decamer-promoting eIF2Bα dimer.

Here, beginning from unbiased efforts to identify eIF2B binders and activators, we showed that sugar phosphates occupy an ancestral ligand pocket in eIF2Bα, promoting holoenzyme decamerization. We present a working model to illustrate that activation of the eIF2B complex can now be achieved in two ways: Nature’s solution, dating back billions of years, and one developed in the last decade through man-made chemistry (Fig. 5d). Perhaps owing to its site of action in eIF2Bα, we found that unlike ISRIB, sugar phosphates do not possess the ability to antagonize the interaction between eIF2B and phospho-eIF2α in a biochemical experiment. Thus, these activators have overlapping but distinct mechanisms.

Although we have demonstrated a functional role for sugar phosphate binding in modulation of eIF2B decamerization and activity, the changes elicited by such an interaction remain unclear as we did not observe major structural shifts in the eIF2Bα structure upon ligand binding. Interpretation of the data are complicated by the fact that a true ligand-free “apo” structure of eIF2Bα does not exist—the solved structures contain a sulfate ion in the sugar phosphate binding pocket (an artifact of the crystallization buffer). Comparisons against existing decameric structures of eIF2B were also uninformative, as those structures already represent the stabilized state of the complex. Elucidation of these changes may require dynamic methods rather than the static “snapshots” obtained herein.

Pathogenic eIF2B partial loss-of-function mutations are present in all subunits. Three of these mutations that map to eIF2Bα provided us with important insights as to the role of sugar phosphates in eIF2B activation in vivo. N208Y and E198K are located in the sugar phosphate binding pocket and both abolish ligand binding. Both mutations had modestly reduced GEF activity and rendered eIF2B unresponsive to sugar phosphates. The knowledge that N208Y is a human disease-causing mutation suggests that occupation of this site may be important for eIF2B function. Similarly, in yeast, eIF2Bα^E199K^ (equivalent to human E198K) was originally identified through screening as a constitutively depressing (i.e. ISR-activating) mutant^34^. An important caveat is that the N208Y mutation (N209Y in yeast) reportedly reduces the level of eIF2Bα in yeast^35^. Whereas eIF2Bα is dispensable for survival in yeast, it is essential in metazoans. This may be one explanation for our inability to generate human eIF2Bα^N208Y^ knock-in mutant cells. Notably, we observed reduced eIF2B subunit levels in HEK293T cells with the eIF2Bα^E198K^ knock-in mutation (Supplementary Fig. 8e). If the N208Y mutation does destabilize eIF2Bα in humans, we cannot deconvolve this effect from an additional effect on metabolite binding. It may also mean that sugar phosphate binding is obligate for the stability of the eIF2Bα subunit. However, clinical evidence implies that eIF2Bα^N208Y^ is viable as the only source of α subunit in a living human^28^, so compensatory mechanisms may exist or the genetic background of HEK293T cells may have been incompatible with this mutation.

In contrast to eIF2Bα^N208Y^, the eIF2Bα^V183F^ mutation disrupts formation of the α_2_ dimer and severely decreases basal GEF activity but is highly responsive to F6P stimulation in vitro. Despite our finding that F6P can partially stabilize this mutant dimer, the pathogenicity of this mutation in vivo suggests that sugar phosphates may not always occupy the pocket in cells. Alternatively, it may mean that ligand binding is insufficient to rescue full eIF2B function in the full physiological context. A recent intriguing observation is the striking cell-type susceptibility of astrocytes to the hypomorphic alleles of eIF2B^13^. The ISR is triggered early on in VWM disease progression only in these cells. It is tempting to speculate that the unique metabolic characteristic of astrocytes may result in lower occupancy by sugar phosphates and overall decreased GEF activity, which could exacerbate the effects of destabilized VWM mutant eIF2B complexes.

Our findings provide a facile explanation for the use of a metabolic enzyme as the eIF2B regulatory core. We propose that sugar phosphate binding may represent an ancient regulatory mechanism that directly couples carbon nutrient sensing to protein synthesis, predating the evolution of the panoply of stress-responsive eIF2α kinases in higher eukaryotes. Intracellular free concentrations of sugar phosphates may vary depending on cell and tissue type, circadian rhythm, nutritional flux, metabolite microdomains and more – these may all impinge on eIF2B function in a physiological setting. It is likely that many cellular systems are governed by similar systems, such as the recently identified role of fructose-1,6-bisphosphate sensing by aldolase as a novel nucleotide-independent mechanism of AMPK regulation^36^. Another well-established allosteric regulator in the cell is fructose-2,6-bisphosphate, which undergoes significant changes in intracellular concentration in response to glucose uptake. We were unable to determine whether this sugar phosphate interacts with eIF2Bα due to lack of a commercial source, but it could be an ideal candidate for a physiological modulator of eIF2B activity.

In yeast, many metabolic enzymes are capable of undergoing dramatic and reversible conformational changes in response to nutrient status. Indeed, yeast eIF2B has been shown to form cell-spanning fibrils, although the conditions for formation and ultimate function of these structures are debated^37,38^. Recent work suggests that eIF2Bα is essential for the formation of these eIF2B bodies, and that VWM mutations (including N209Y) disrupt this process^39^. We thus speculate that metabolite binding may be one possible mechanism to modulate these conformational shifts. It is conceivable that many similar mechanisms existed to support the most basic needs of early unicellular organisms—to grow when resources are abundant, and to quiesce during periods of scarcity. Our observation here that an eIF2Bα mutation abrogates its nutrient sensing ability and causes VWM disease in humans suggests the tantalizing possibility that this function may retain some importance even in complex multicellular organisms. It is possible that the pocket occupied by synthetic eIF2B activators may also serve as a binding site for as-yet-unidentified naturally occurring metabolites. This pocket may sense metabolites involved in a different pathway to couple protein synthesis capacity to other processes in the cell. Ultimately, understanding the role of ligand binding in control of eIF2B activity in vivo will be of utmost importance. Our in vitro data lay the groundwork for addressing these questions using more complex experimental systems.

## Methods

### Protein purification

The nucleotide sequence of eIF2Bα (Uniprot accession: Q14232) was synthesized by Genscript with a C-terminal TEV-avi-FLAG tag and cloned into a pET45b vector (Novagen) for expression. Point mutations of eIF2Bα were introduced by site-directed mutagenesis. All constructs were sequenced to verify the expected sequences.

Wild-type eIF2Bα and the mutants (V183F, E198K, N208Y) were expressed in BL21(DE3) cells (NEB). Cells were grown to a density of OD600 = 0.8 and induced with 360 μM IPTG. Cells were shaken in a beveled flask at 200 rpm, 18 °C for 16 h. Cells were harvested by pelleting and resuspended in 25 mM Tris, 150 mM NaCl, 1 mM DTT, pH 8.0 + Halt protease inhibitor cocktail (Thermo Fisher). Cells were lysed by sonication and lysates were cleared by centrifugation at 33,000 × *g*, 4 °C, 1 h. Supernatants were purified by FLAG affinity chromatography (Genscript). Target fractions were collected, and proteins were further purified on a Superdex200 column (GE Healthcare) using 20 mM HEPES, 150 mM NaCl, 1 mM DTT, pH 7.5. Purified proteins were concentrated and stored at −80 °C until further use. Purification of eIF2B(βδγε) and eIF2 was performed, as previously described^12^.

### Compounds and antibodies

ISRIB was synthesized in-house as previously described^40^. Metabolites were purchased from Millipore Sigma, Cayman Chemicals, Avanti Polar Lipids, Enamine or Combi-Blocks, Inc and prepared as 10 mM stocks arrayed in 96-deep well storage plates and stored at −80 °C. Information on source and solvent for each metabolite is available in Supplementary Data 1 and 2. Metabolite accurate mass, adduct, purity, and optimal detection parameters were determined by independently assaying each metabolite by FIA-MS at pH 3, 5, 6.8, and 9 in positive and negative mode (4 technical replicates) with interspersed blank injections on an Agilent 6550 QTOF MS platform. The optimal adduct, pH, and polarity of each metabolite was considered to construct four unique metabolite screening pools validated by FIA-MS on Agilent 6550 QTOF MS and SCIEX X500R QTOF MS platforms.

The following antibodies were used in this study: eIF2Bα (Proteintech #18010–1-AP); eIF2Bδ (Proteintech #11332–1-AP); eIF2Bε (Bethyl Labs #A302-556); eIF3a (Cell Signaling Technology #3411); eIF2α (Cell Signaling #5324); p-eIF2α (Cell Signaling #3398); FLAG (Sigma #F1804). All antibodies were used at 1:1000 dilution for detection.

### MIDAS

Metabolites were combined into four defined screening pools. For each metabolite pool, 5 µL of ~960 µM eIF2Bα was arrayed in triplicate across a 10 MWC 96-well microdialysis plate (SWISSCI) and sealed (protein chamber). To the reverse side, 300 μL of 50 μM per metabolite pool was aliquoted and sealed (metabolite chamber). Loaded dialysis plates were placed in the dark at 4 °C on a rotating shaker (120 rpm) and incubated for 40 h. Post-dialysis, metabolites were isolated from each chamber by cold methanol extraction. Each sample was analyzed in technical triplicate by FIA-MS on a SCIEX X500R QTOF MS with interspersed blanks injections. The input metabolite pools were assayed at the beginning, middle, and end of each MS method batch for QC and normalization.

MIDAS FIA-MS spectra were processed in SCIEX OS 1.6 software to determine metabolite abundances by integrating the mean area under the curve for each extracted ion chromatogram. For each dialysis replicate, log_2_(fold change) for each metabolite was calculated as the difference between the log_2_ abundance in the protein chamber and metabolite chamber. For each technical triplicate, up to one outlier was removed using a z-score cutoff of five (<0.1% of observations). The corrected technical replicates were collapsed to one mean fold-change summary per protein-metabolite pair. To remove fold-change variation that was not specific to a given metabolite-protein pair, the first three principal components of the total screening dataset were removed on a per metabolite pool basis by subtracting the projection of the first 3 principal components, creating log_2_(corrected fold change). Protein-metabolite z-scores were determined by comparing the target protein-metabolite log_2_(corrected fold change) to a no-signal model for that metabolite using measures of the central tendency (median) and standard deviation (extrapolated from the 25–75% quantiles), which are robust to the signals in the tails of a metabolite’s fold-change distribution. Z-scores were false-discovery rate controlled using Storey’s q-value (qvalue, R) and protein-metabolite interactions with *q*-values < 0.1 were considered significant.

### Differential scanning fluorimetry

The effect of metabolite binding on eIF2Bα T_m_ was assessed using the Protein Thermal Shift assay (Thermo Fisher), following manufacturer’s instructions. Briefly, reaction conditions for each 20 μL reaction were: 1.1 μg (1.5 μM) of eIF2Bα, 5 μl Thermal Shift buffer, 1X Thermal Shift dye, and metabolites in dose response. Reactions were run in 384-well format on a QuantStudio 6 Flex instrument (Thermo Fisher). The resulting melt curves were analyzed using Protein Thermal Shift Software, and eIF2Bα T_m_s for each condition were calculated by fitting to the Boltzmann function.

### Isothermal titration calorimetry

Interactions between recombinant protein and sugar phosphates were conducted with a MicroCal PEAQ-ITC (Malvern Panalytical Inc.). Experiments were performed in 25 mM HEPES, 150 mM NaCl, pH 7.5 at 20 °C. The sugar phosphate concentration in the syringe was 1 mM or 1.5 mM, and ~3.5 μL aliquots were injected into cells containing 300 μL eIF2Bα or eIF2B(βδγε) at a concentration of 20–30 μM. The final titration curves were fitted using MicroCal ITC data analysis software, assuming a single binding site per monomer.

### eIF2B activity assays

Bodipy-FL-GDP-loaded eIF2 was used as a substrate for recombinant eIF2B. The assay was performed in 384-well plates. Reactions were read on a SpectraMax i3x plate reader (Molecular Devices) using the instrument’s Softmax Pro software with the following parameters: plate temperature = 25 °C; excitation wavelength = 485 nm (15 nm width); emission wavelength = 535 nm (25 nm width); read duration = 30 min at 45 s intervals. Data were analyzed in Prism. GDP release half-lives were calculated by fitting single-exponential decay curves.

For unbiased screening, MIDAS metabolite libraries at 10 mM stock concentration were dispensed into wells of the assay plate using an Echo Acoustic Liquid Handler (Labcyte). In a final assay volume of 10 μL/well, the following conditions were kept constant: 25 nM Bodipy-FL-GDP-loaded eIF2, 4 nM phospho-eIF2, 0.1 mM GDP, 1 mg/mL BSA, 1 nM eIF2B, 10 μM metabolite. A single well was run for each metabolite. For targeted experiments with recombinant wild-type and mutant eIF2Bα, the following conditions were used: 50 nM Bodipy-FL-GDP-loaded eIF2, 0.1 mM GDP, 1 mg/ml BSA, 20 nM eIF2B. For targeted experiments with HEK293T lysates, the following conditions were used: 50 nM Bodipy-FL-GDP-loaded eIF2, 0.1 mM GDP, 1 mg/ml BSA, 0.1 or 0.3 mg/ml lysates.

### Cryo-EM data collection and image processing

eIF2Bα-F6P and eIF2B(βδγε) were mixed in a ~1.5:1 molar ratio and incubated on ice for 1 h. Prior to grid preparation, the protein mixture was diluted with 25 mM HEPES, 100 mM KCl, 2 mM MgCl_2_, 1 mM DTT, pH 7.5 to a final concentration of ~0.3 mg/mL.

A 3 μL drop of the sample was applied to a 1.2/1.3 UltrAuFoil grid (Quantifoil) that had been plasma-cleaned for 10–40 s using a 25% O_2_/75% Ar mixture in a Solarus 950 Plasma Cleaner (Gatan). Grids were plunge-frozen in liquid ethane using the following settings on a Vitrobot Mark IV (Thermo Fisher Scientific): blot time 4–6 s, 4 °C, 100% humidity. The grids were imaged using an FEI Titan Krios (Hillsboro, Oregon) transmission electron microscope operated at 300 kV and equipped with a Volta phase plate and Gatan K2 Summit direct detector placed at the end of a GIF Quantum 967 LS imaging filter, operating with a slit width of 20 eV (Gatan, Inc.). Automated data collection was performed with Leginon software at a nominal magnification of ×130,000, corresponding to a pixel size of 1.04 Å^41^. A total of 4506 movies were recorded using a nominal defocus range of −1.0 to −2.4 μm. Exposures were fractionated into 30 frames with an exposure rate of 7.6 e^–^/pixel/s and total exposure of 44.3 e^–^/Å^2^. An additional dataset of 1567 movies was acquired at 30° tilt^42^. Data collection and image processing parameters for tilted and untilted data were identical.

Movie frames were motion-corrected and dose-weighted using MotionCor2^43^. Further image processing was carried out in cryoSPARC^44^. CTF parameters were estimated from the dose-weighted aligned movie frames with Patch CTF. The images acquired at 0° and 30° tilt were pooled together. Projections of the cryo-EM structure of *H. sapiens* eIF2B decamer (EMD-7443)^10^ were generated using EMAN and were subsequently used as templates for particle picking. From 6073 total images collected, 1865 were rejected prior to 2D/3D classification in cryoSPARC. The 736,044 particles from the remaining 4208 images were subjected to both 2D classification and 3D heterogenous refinement simultaneously. Multiple rounds of heterogeneous and homogeneous refinement were performed using the eIF2B map low-pass filtered to 25 Å as an initial reference. During each round of 3D classification, only one of the models appeared to have the correct size corresponding to the intact eIF2B complex, whereas the other classes were too small to be the complex of interest. This 3D class had clear high-resolution features and was directly refined from that point, obviating the need for 2D classification for particle sorting. This was followed by several additional rounds of heterogeneous refinement and non-uniform refinement with C2 symmetry imposed. Subsequently, the map was refined by two rounds of global and per-particle CTF optimization, resulting in a final map with a resolution of 2.9 Å using the gold-standard FSC = 0.143 criteria.

Rigid-body docking of the coordinates of the *H. sapiens* eIF2B (PDB: 6CAJ)^10^ into the reconstructed EM density map showed reasonably good fit. Several rounds of manual model adjustment in COOT and refinement in phenix.real_space_refine were applied. The initial maps revealed noticeable variability in map resolution, showing the highest resolution at the center of the complex (for eIF2Bα/β/δ) and much lower resolution at the periphery of the complex. The resolution was lowest for the eIF2Bγ/ε subunits, suggesting a higher amount of disorder/flexibility in this region. During the model building and refinement phase, a small density feature corresponding to F6P was observed only in the eIF2Bα subunit in the area that M6P has been previously modeled in the X-ray crystal structure. Analysis and model validation were performed with the aid of MOLPROBITY and COOT and Phenix validation tool. Data collection and processing statistics are provided in Supplementary Table 1.

### Crystallization and structure determination

The avi-FLAG tag on eIF2Bα was removed by TEV protease (NEB) prior to crystallization. For co-crystallization, eIF2Bα was incubated with 8 mM mannose-6-phosphate on ice for 1 h. Crystals of eIF2Bα in complex with mannose-6-phosphate were grown by sitting-drop vapor diffusion method at 18 °C for 5 days. The protein complex (~5 mg/ml) was crystallized by mixing 1.5 μL of the protein solution with 1.5 μL of reservoir solution (12% PEG 4000, 100 mM sodium acetate, 100 mM ammonium sulfate, 0.5% octyl-beta-glucoside, pH 4.6), and equilibrated over 360 μL of the reservoir solution.

Crystals belonged to monoclinic space group P21 with the following unit cell parameters: *a* = 71.2 Å, *b* = 155.5 Å, *c* = 140.11 Å, and α = γ = 90°, β = 103.9°. Prior to data collection, crystals were cryo-protected by addition of 25% glycerol (final concentration) to the crystallization drop. X-ray diffraction data were collected on the IMCA beamline at beamline 17-ID in the facilities of the Industrial Macromolecular Crystallography Association Collaborative Access Team (IMCA-CAT) at the Advanced Photon Source, Argonne National Laboratory. Data were reduced and scaled using autoPROC software. A previously reported eIF2Bα structure (PDB: 3ECS) was used as a starting model for iterative rounds of map fitting and refinement using the programs COOT, Buster, Refmac and Phenix. Data collection and refinement statistics are provided in Supplementary Table 2.

### Generation of mutant cell lines

The eIF2Bα^E198K^ mutation was introduced into HEK293T cells using the Alt-R CRISPR-Cas9 System (IDT). cRNA sequences were chosen with the IDT design tool to allow for CRISPR cutting near the E198 site. To introduce the point mutation, a 150-bp DNA donor template for homology-directed repair (HDR) was synthesized to include the G > A point mutation of interest, as well as silent mutations to prevent further Cas9 cutting.

To form CRISPR gRNA complex, equimolar amounts of crRNA and tracrRNA labeled with Atto550 were annealed by heating to 95 °C for 5 min followed by cooling to room temperature. RNP complex was formed by incubating gRNA complex with Alt-R S.p. HiFi Cas9 Nuclease V3 (IDT). RNP complex, 100 μM HDR template, and 100 μM Alt-R Cas9 Electroporation Enhancer (IDT) were delivered to cells with a Lonza 4D-Nucleofector, using the 96-well SF reagent kit and program SF-130 following vendor instructions. Nucleofected cells were incubated in media containing 30 μM Alt-R HDR Enhancer (IDT). After 48 h, Atto550-positive cells were single-sorted into 96-well plates using a BD Biosciences FACSAria Fusion. Clones were expanded and genomic DNA was sequenced to confirm introduction of the mutant allele. A similar protocol was employed to generate HEK293T cells with eIF2Bβ tagged with a C-terminal FLAG peptide at the endogenous locus. The eIF2Bα^V183F^ HEK293T cell line was previously described^12^.

### Sucrose gradients

WT or eIF2Bα mutant HEK293T cells were harvested and lysed in 50 mM HEPES, 400 mM KCl, 1 mM DTT, 4 mM Mg(OAc)_2_, pH 7.5 + 0.5% Triton-X-100 + EDTA-free protease inhibitor cocktail (Roche) on ice for 30 min. Lysates were cleared by centrifugation at 48,000 × *g*, 4 °C for 30 min. 100 μL of each cleared lysate was loaded onto a 5–20% sucrose gradient prepared in a 13 × 51 mm ultracentrifuge tube using a Gradient Station (Biocomp) and centrifuged in a SW55 Ti rotor at 152,000 × *g*, 4 °C for 12 h. After centrifugation, 24 × 200 μL fractions were manually collected from the top to bottom of each gradient. Each fraction was subjected to methanol/chloroform precipitation and SDS-PAGE followed by Western Blot with the indicated antibodies. Blots were imaged on a ChemiDoc MP (Bio-Rad), and band intensities were quantified using the instrument’s Image Lab software. Each value was then divided by total intensity obtained across all fractions to determine percent enrichment of each subunit in each fraction.

### Size-exclusion chromatography

Purified proteins were thawed and centrifuged at 20,000 × *g*, 10 min, 4 °C to remove any potential precipitate. Concentrations of eIF2B(βδγε) and eIF2Bα were normalized to 2 μM and 2.4 μM, respectively. 0.2 mL of each mixture was injected onto a Superose 6 Increase 10/300 column connected to an AKTA Pure 25 FPLC system (GE Healthcare). The system was run at 0.5 mL/min for 1 h using 25 mM HEPES, 200 mM KCl, 2 mM MgCl_2_, 1 mM DTT, pH 7.5 as the mobile phase. For conditions with ISRIB, the protein samples and mobile phase were supplemented with 500 nM ISRIB. For conditions with F6P, the protein samples and mobile phase were supplemented with 200 μM F6P. UV_280_ measurements were obtained directly by the instrument.

### Phospho-eIF2α co-immunoprecipitation

HEK293T cells endogenously expressing eIF2Bβ with a C-terminal FLAG tag were maintained in DMEM High Glucose (Corning CellGro) supplemented with 10% FBS (Gibco) and antibiotic-antimycotic (Gibco). Upon reaching 90% confluency, cells were treated with either DMSO or 100 nM thapsigargin (Sigma) for 4 h. Cells were harvested and lysed in 25 mM HEPES, 150 mM KCl, 1% NP-40, 1 mM EDTA, pH 7.4 + Complete protease inhibitor cocktail (Roche) and PhosStop (Roche) on ice for 20 min. Lysates were cleared by centrifugation at 18,000 ×  *g*, 20 min, 4 °C, and transferred to Eppendorf tubes containing anti-FLAG M2 magnetic beads (Sigma). Mixtures were incubated overnight at 4 °C. To test the effect of ligands, these were added to the mixtures and incubated for an additional 2 h at 4 °C. Beads were washed three times with lysis buffer, eluted with 1× LDS buffer (Invitrogen), and subjected to SDS-PAGE followed by Western Blot with the indicated antibodies.

### Reporting summary

Further information on research design is available in the Nature Research Reporting Summary linked to this article.

## Supplementary information

Supplementary Information

Supplementary Data 1

Supplementary Data 2

Description of additional supplementary files

Reporting Summary

## Data Availability

The data generated or analyzed in the current study are available within the article, supplementary information, or from the corresponding authors upon reasonable request. The structures reported in this work have been deposited in public repositories with the following accession codes: eIF2B-F6P cryo-EM structure: PDB 7KMF, EMDB EMD-22924; eIF2Bα-M6P X-ray crystal structure: PDB 7KMA. Source data are provided with this paper.

## Source data

source data
