# Supplementary material for: Sugar phosphate activation of the stress sensor eIF2B
Source: Nat Commun. 2021 Jun 8;12:3440. doi: 10.1038/s41467-021-23836-z (PMC8187479; doi:10.1038/s41467-021-23836-z)
Supplement: Supplementary file 1 — Supplementary Information [file 41467_2021_23836_MOESM1_ESM.pdf]

SUPPLEMENTARY INFORMATION

Sugar phosphate activation of the stress sensor eIF2B

Qi Hao[1], Jin-Mi Heo[1,2], Boguslaw P. Nocek[3], Kevin G. Hicks[4], Vincent S. Stoll[3], Clint Remarcik[1], Sean Hackett[1], Lauren LeBon[1], Rinku Jain[3], Dan Eaton[1], Jared Rutter[4,5], Yao Liang Wong[1]*, Carmela Sidrauski[1]*

[1] Calico Life Sciences LLC, South San Francisco, CA, USA

[2] Present address: Loxo Oncology at Lilly, South San Francisco, CA, USA

[3] Research & Development, AbbVie, 1 North Waukegan Road, North Chicago, IL, USA

[4] Department of Biochemistry, University of Utah School of Medicine, Salt Lake City, UT, USA

[5] Howard Hughes Medical Institute, University of Utah School of Medicine, Salt Lake City, UT, USA

These authors contributed equally: Qi Hao and Jin-Mi Heo

These authors jointly supervised this work: Yao Liang Wong and Carmela Sidrauski

* Correspondence: yao@calicolabs.com, carmela@calicolabs.com

CONTENTS

Supplementary Figures 1-11.

Supplementary Tables 1-2.

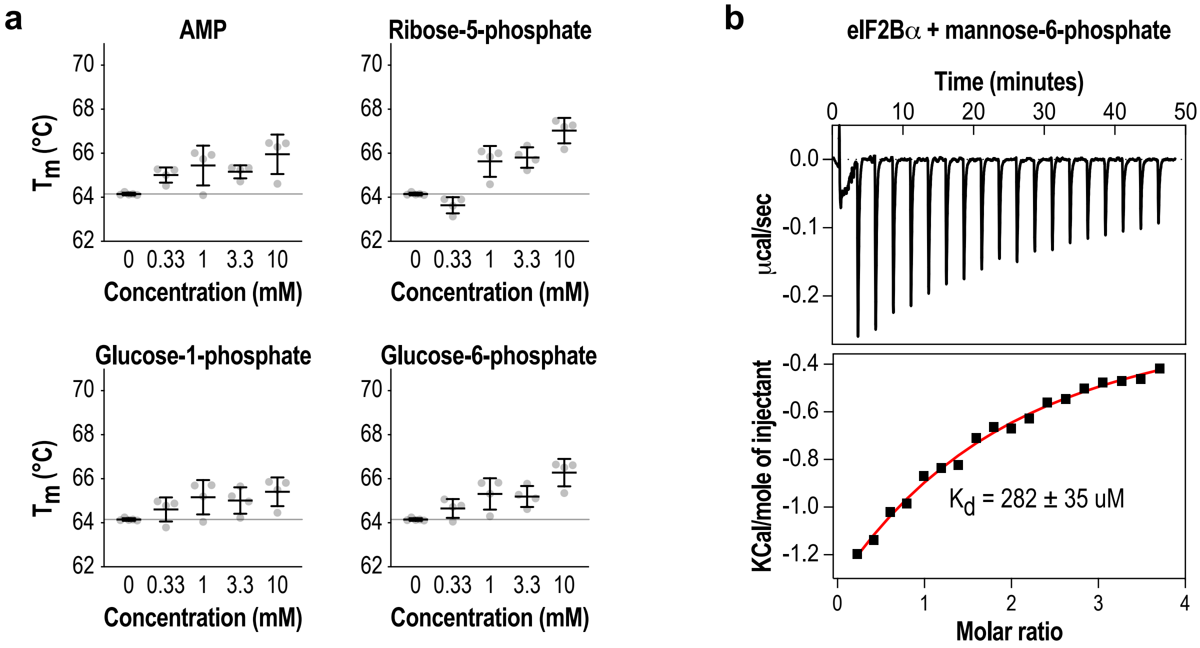

Supplementary Figure 1. **Interactions of eIF2Bα with sugar phosphates. a** Differential scanning fluorimetry of eIF2Bα in combination with selected metabolites in dose-response. Metabolite binding increased the T_m of eIF2Bα. Bars are mean ± standard deviation of n=4 independent experiments. **b** K_d of the eIF2Bα-M6P interaction measured by ITC. The upper subpanel shows the baseline-subtracted thermogram. The bottom subpanel represents the binding isotherm, with the red line indicating the fit curve.

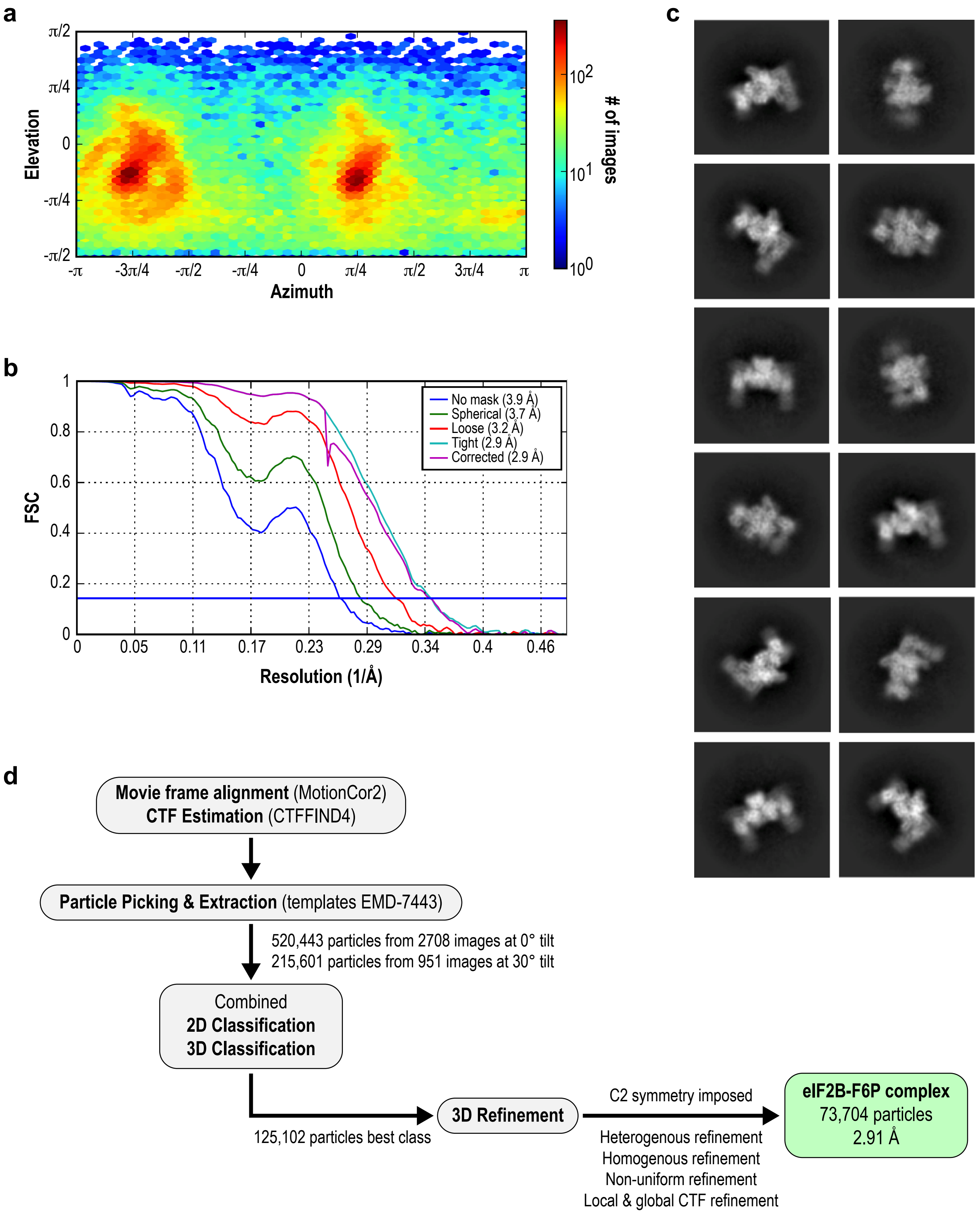

Supplementary Fig. 2. **Structure and resolution determination of eIF2B-F6P. a** Per-particle distribution over azimuth and elevation angles using cryoSPARC v2. **b** Gold-standard Fourier shell correlations for the final density map, with a resolution of 2.9 Å. **c** Samples of 736,044 particle images selected by template matching after 2D classification, acquired at different orientations and 0° or 30° tilt. **d** Flowchart summary of cryo-EM processing steps from frame alignment to 3D reconstruction. C2 symmetry was imposed during homogenous refinement after two rounds of 3D classification and at all subsequent refinement steps.

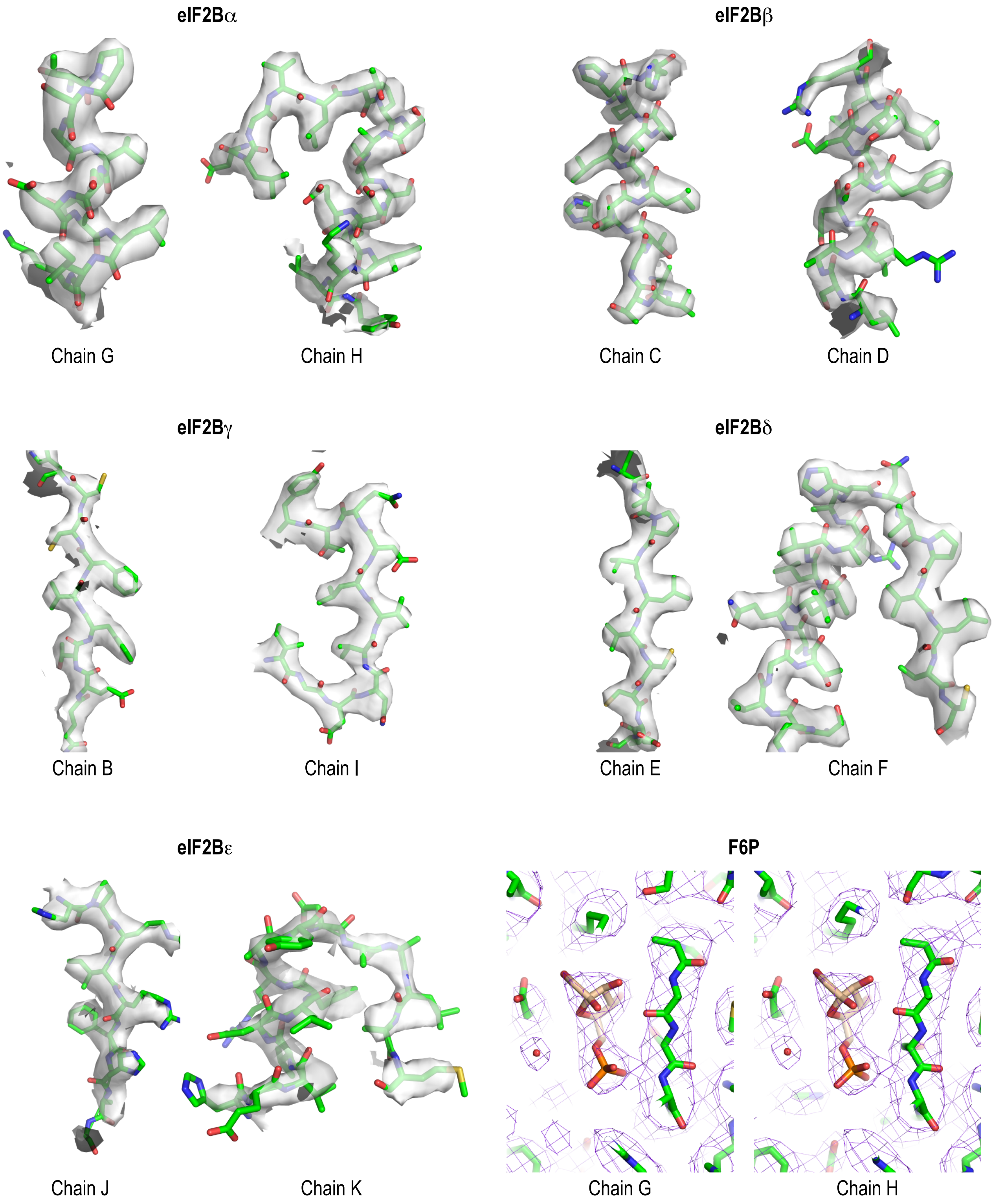

Supplementary Figure 3. **Cryo-EM density maps for selected regions of eIF2B.** Cryo-EM density is depicted by the gray surface and the atomic model as green sticks. Density for F6P bound to the eIF2Bα subunits is shown in the bottom right. Density for the two eIF2Bε subunits is significantly lower resolution than the overall value of 2.9 Å. Cryo-EM density for F6P and neighboring amino acids is well-resolved, allowing placement of a few solvent molecules (red spheres).

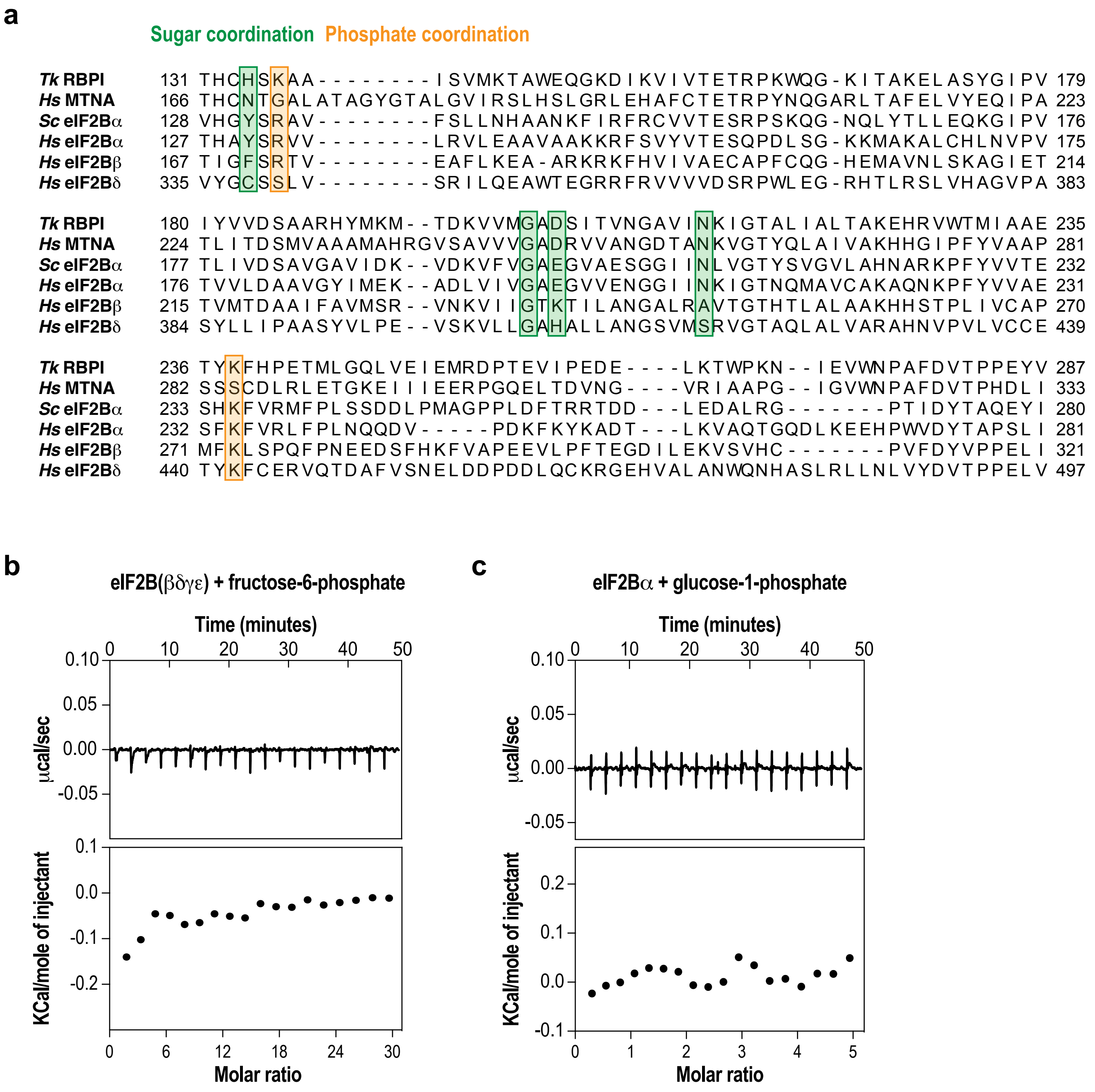

Supplementary Figure 4. **Sequence alignment of eIF2Bα and its homologs supports the structural model of residues critical for metabolite binding. a** Protein sequences of human eIF2Bα, eIF2Bβ and eIF2Bδ were aligned with human methylthioribose-1-phosphate isomerase (MTNA), *T. kodakarensis* ribose-1,5-bisphosphate isomerase (RBPI) and *S. cerevisiae* eIF2Bα using MUSCLE. Only the region immediately encompassing the eIF2Bα ligand-binding pocket is shown. Sugar-coordinating and phosphate-coordinating residues based on the eIF2B-F6P structure are indicated in green and orange, respectively. **b** eIF2B(βδγε) does not interact with F6P, as measured by ITC. **c** eIF2Bα does not interact with G1P, as measured by ITC. In **b** and **c**, the upper subpanel shows the baseline-subtracted thermogram. The bottom subpanel represents the binding isotherm. No curves were fit due to lack of detectable binding.

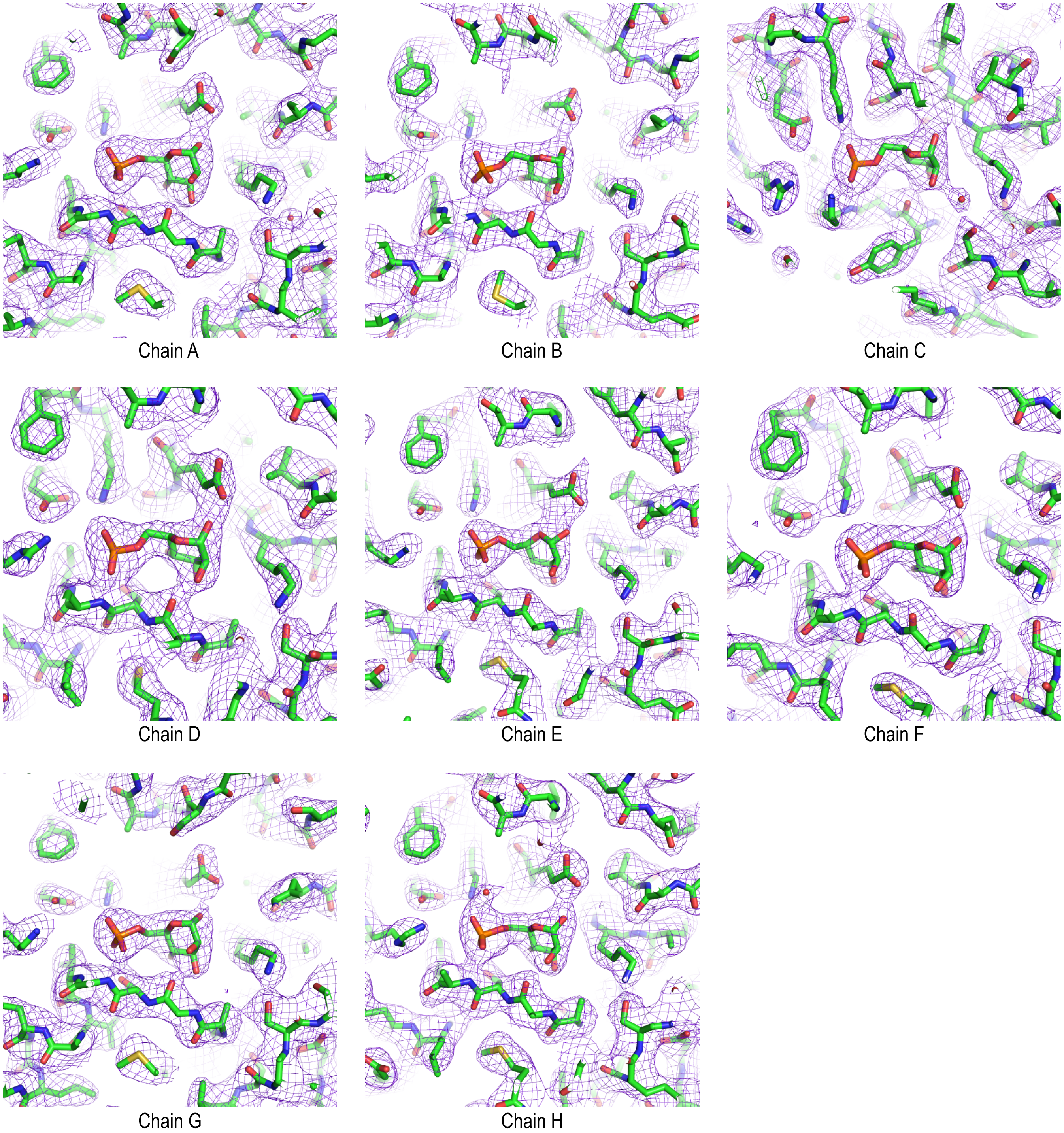

Supplementary Figure 5. **2Fo-Fc density for M6P bound to eIF2Bα.** Density is shown at 1σ (purple mesh) for all chains in the asymmetric unit of the unit cell.

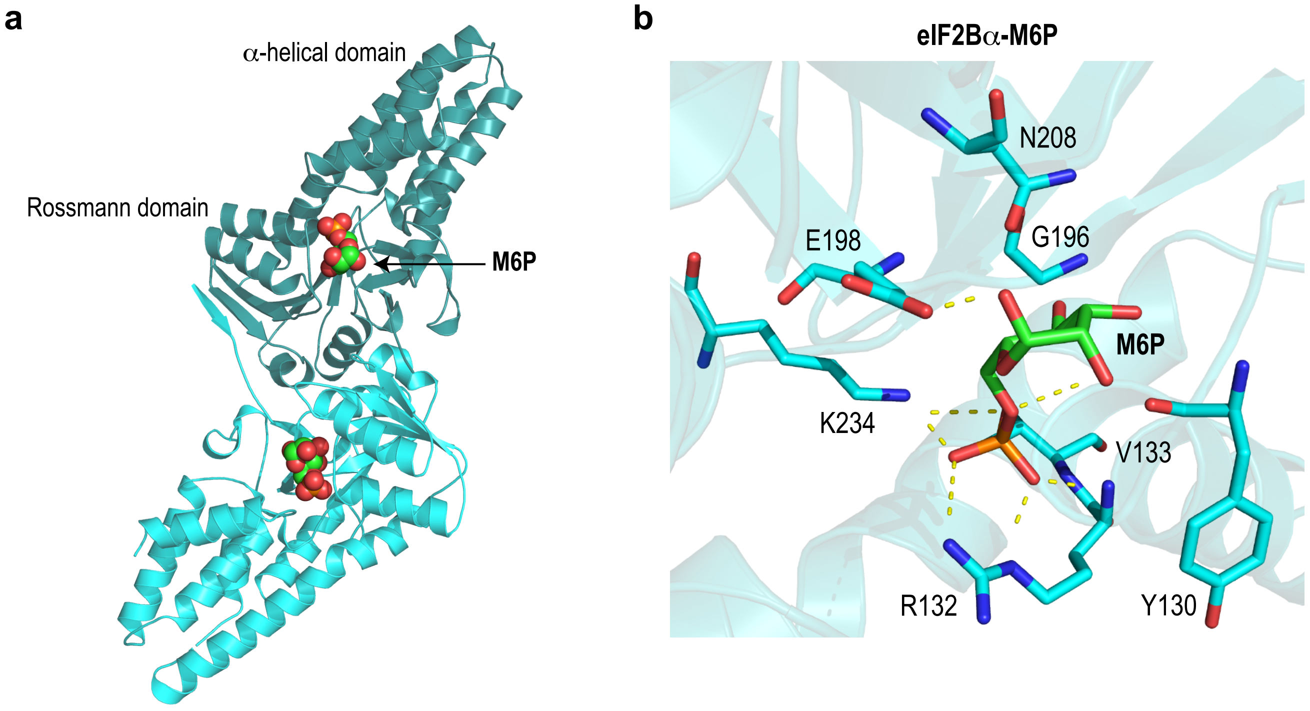

Supplementary Figure 6. **2.7 Å X-ray crystal structure of eIF2Bα in complex with M6P (PDB 7MKA). a** Overall structure of the eIF2Bα-M6P complex, with each protomer bound to one molecule of M6P. One eIF2Bα protomer is colored teal and the second protomer is colored cyan. The α-helical domain and the Rossmann fold-like domain are indicated. M6P is shown as space-filling spheres. **b** M6P recognition through H-bonds contributed by surrounding residues. M6P is shown as green sticks. The residues coordinating M6P are represented as cyan sticks, with H-bonds represented by dashed yellow lines.

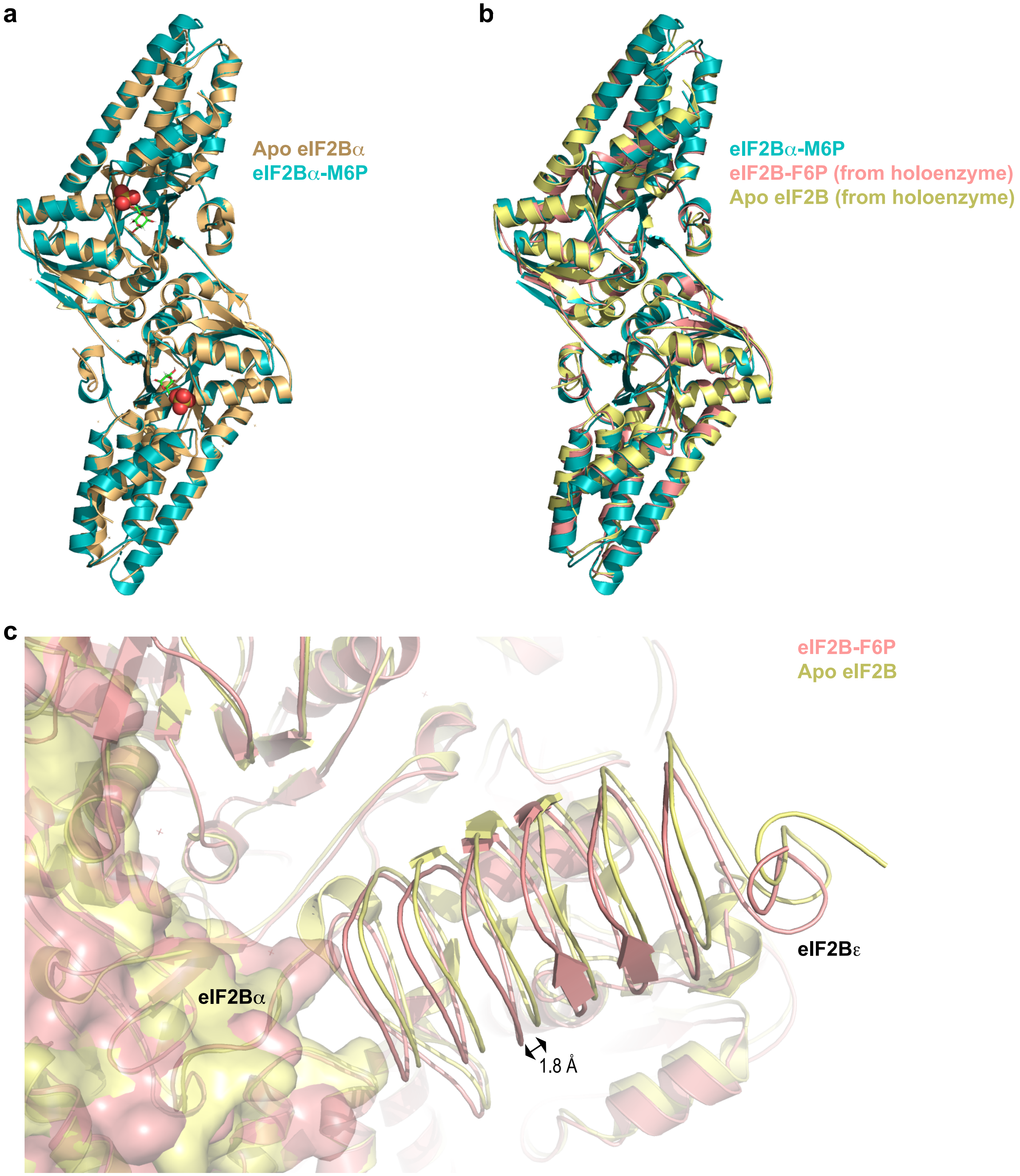

Supplementary Figure 7. **Superposition of sugar phosphate-bound eIF2B structures with apo structures. a** Apo eIF2Bα (PDB 3ECS) aligned to our structure of M6P-bound eIF2Bα (PDB 7KMA) with rmsd = 0.44 Å. M6P is shown in stick representation and SO_4 molecules occupying the sugar phosphate binding site in the apo structure are shown as spheres. **b** M6P-bound eIF2Bα aligned to eIF2Bα from the holoenzyme structures (our F6P-bound structure; PDB 7KMF and the apo holoenzyme; PDB 7D46) with rmsd < 0.9 Å. **c** Zoomed-in view of the β-sheet rich domain of one eIF2Bε subunit, showing an inward movement towards the eIF2Bα subunit (shown in surface representation) in the F6P-bound structure.

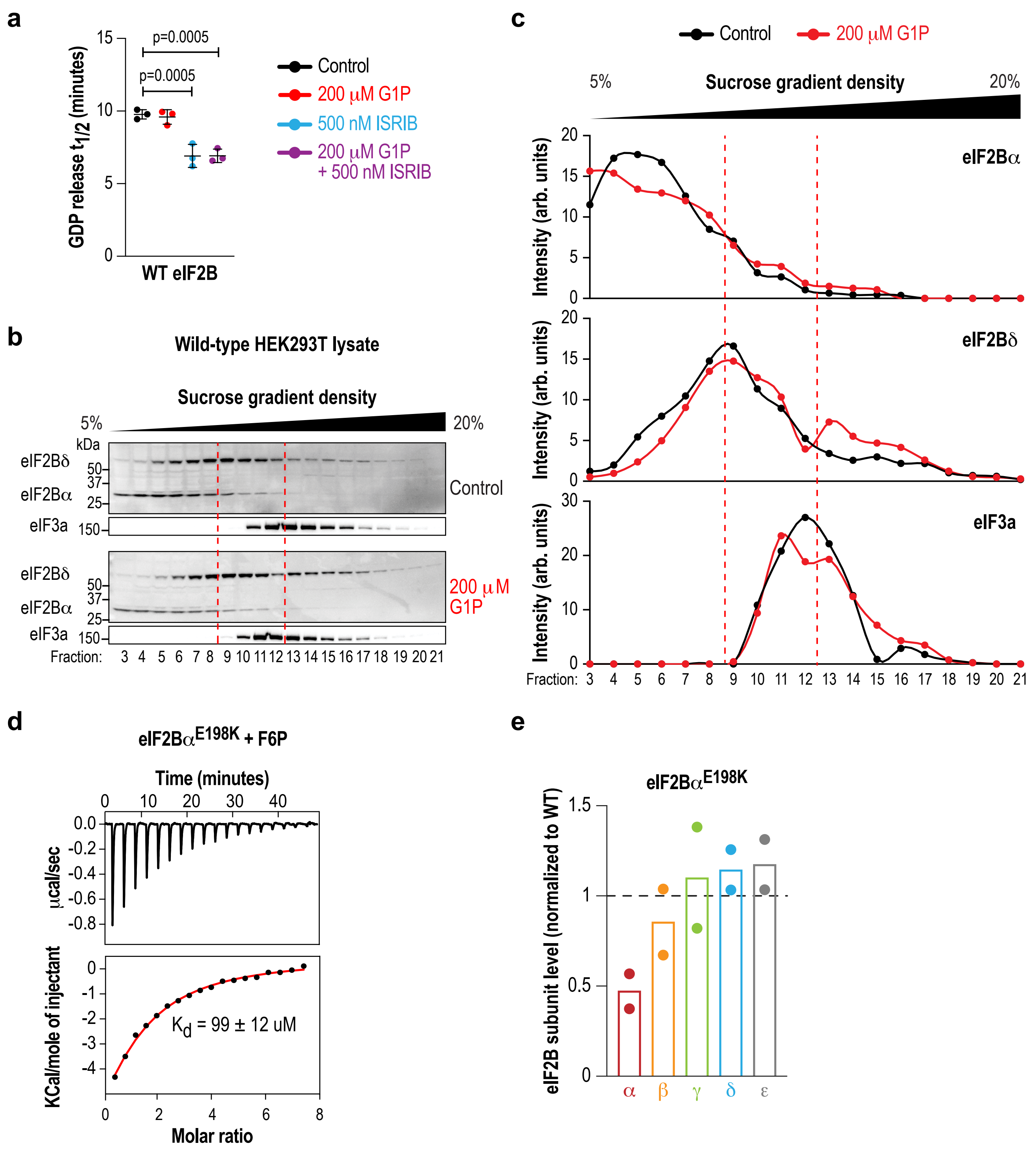

Supplementary Figure 8. **Sugar phosphate identity dictates its ability to enhance eIF2B decamer formation and activity. a** GDP release $t_{1/2}$ in a GEF assay using recombinant wild-type eIF2B. Activity is stimulated by ISRIB (red), but not G1P (blue). Bars are mean ± standard deviation of n=3 independent experiments of 3 technical replicates each. Statistical significance was tested by one-way ANOVA with Dunnett's multiple testing correction. **b** eIF2B complex assembly from wild-type HEK293T lysate treated with G1P was monitored by sucrose gradient centrifugation. Fractions from the sucrose gradient were subjected to SDS-PAGE followed by immunoblotting with the indicated antibodies. eIF3a was used as an internal control. **c** Quantification of **b** normalized by total intensity of each subunit in its respective gradient. Data shown are representative of 2-3 replicates of each experiment. In **b** and **c**, dashed red lines demark the boundary of the decameric eIF2B peak. G1P did not promote eIF2B decamer formation. **d** K_d of the eIF2Bα^{E198K}-F6P interaction measured by ITC. The upper subpanel shows the baseline-subtracted thermogram. The bottom subpanel represents the binding isotherm, with the red line indicating the fit curve. **e** Antibody-based quantification of eIF2B subunits from HEK293T lysates of wild-type and eIF2Bα^{E198K} cells. For each subunit, the signal was normalized to wild-type subunit levels. Data from 2 biological replicates are shown.

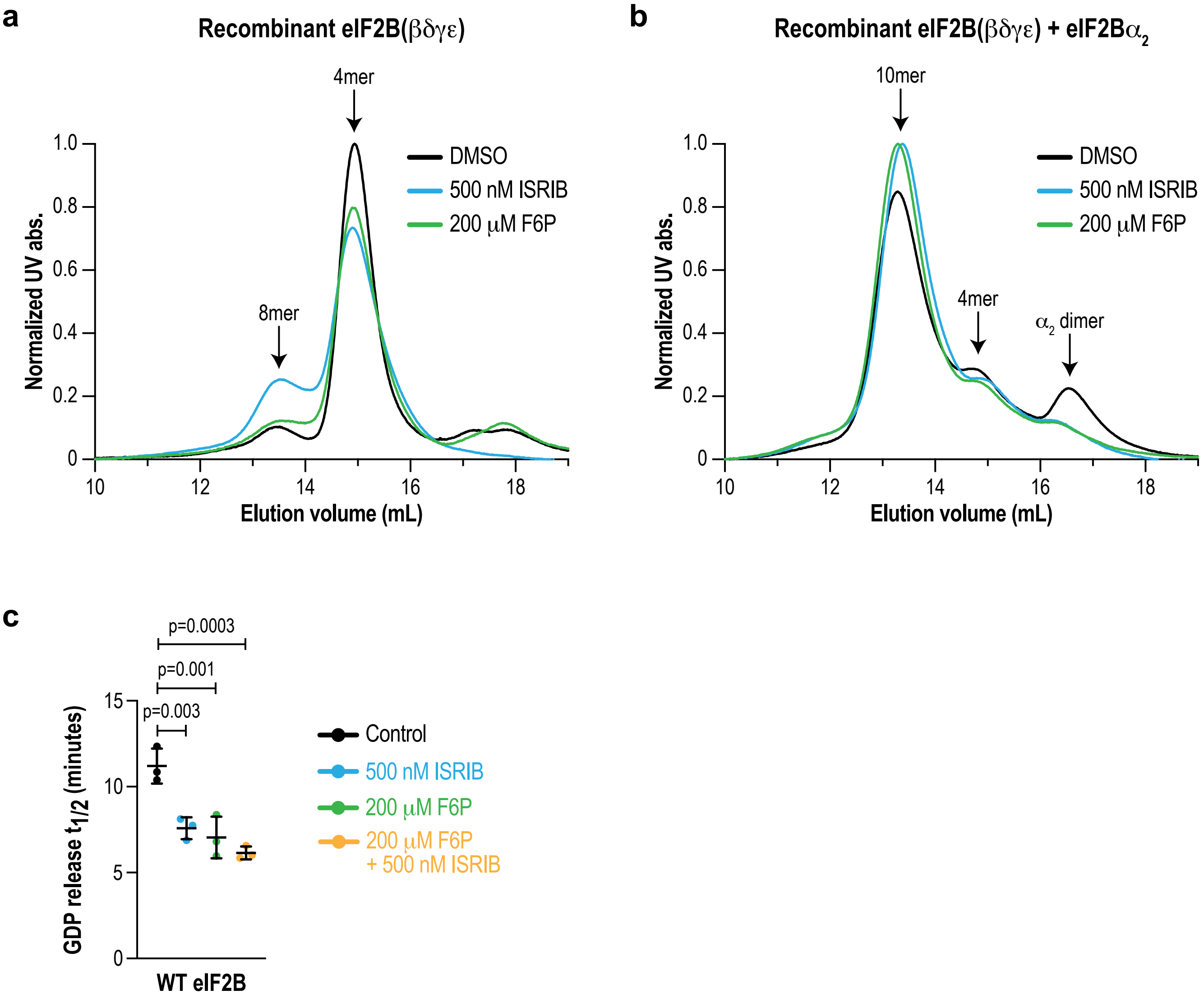

Supplementary Figure 9. **F6P only enhances eIF2B complex stability in the presence of eIF2Bα, whereas ISRIB functions in the absence of eIF2Bα. a** Size-exclusion chromatograms of purified recombinant eIF2B($\beta\delta\gamma\varepsilon$) in the absence or presence of ISRIB (blue) or F6P (green). The elution positions of the $(\beta\delta\gamma\varepsilon)_2$ octamer and ($\beta\delta\gamma\varepsilon$) tetramer are indicated. **b** Size-exclusion chromatograms of purified recombinant eIF2B($\beta\delta\gamma\varepsilon$) + eIF2B$\alpha_2$ in the absence or presence of ISRIB or F6P. The elution positions of the $(\alpha\beta\delta\gamma\varepsilon)_2$ decamer, ($\beta\delta\gamma\varepsilon$) tetramer and α_2 dimer are indicated. In **a** and **b**, traces are representative examples of at least 2 experiments. **c** GDP release $t_{1/2}$ in a GEF assay using recombinant wild-type eIF2B. Activity is stimulated by ISRIB and F6P, but the combination of both (orange) is not additive. Bars are mean ± standard deviation of n=3 independent experiments of 3 technical replicates each. Statistical significance was tested by one-way ANOVA with Dunnett's multiple testing correction.

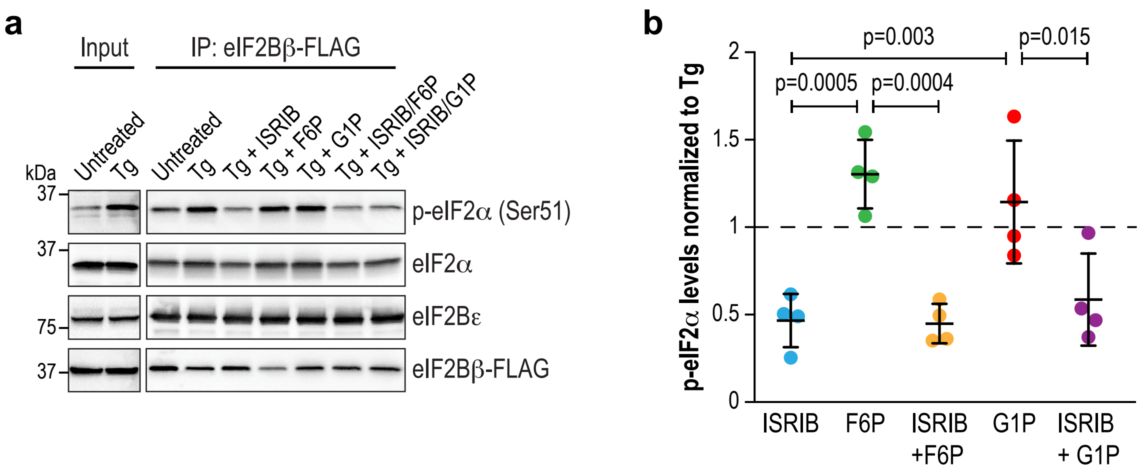

Supplementary Figure 10. **ISRIB, but not F6P, antagonizes p-eIF2α binding to eIF2B. a** Co-immunoprecipitation of phospho-eIF2α from HEK293T cells endogenously expressing FLAG-tagged eIF2Bβ and stressed with thapsigargin (Tg). Ligand incubations were performed at 4°C for 2 hours. Immunoprecipitation was performed against the FLAG epitope. Western Blots of the indicated proteins are shown. **b** Quantification of co-immunoprecipitation experiment. Densitometric measurements were abundance-normalized to eIF2Bβ-FLAG, then normalized again to the Tg condition. Each bar represents mean ± standard deviation of n=4 independent experiments. Statistical significance was tested by one-way ANOVA with Sidak's multiple testing correction.

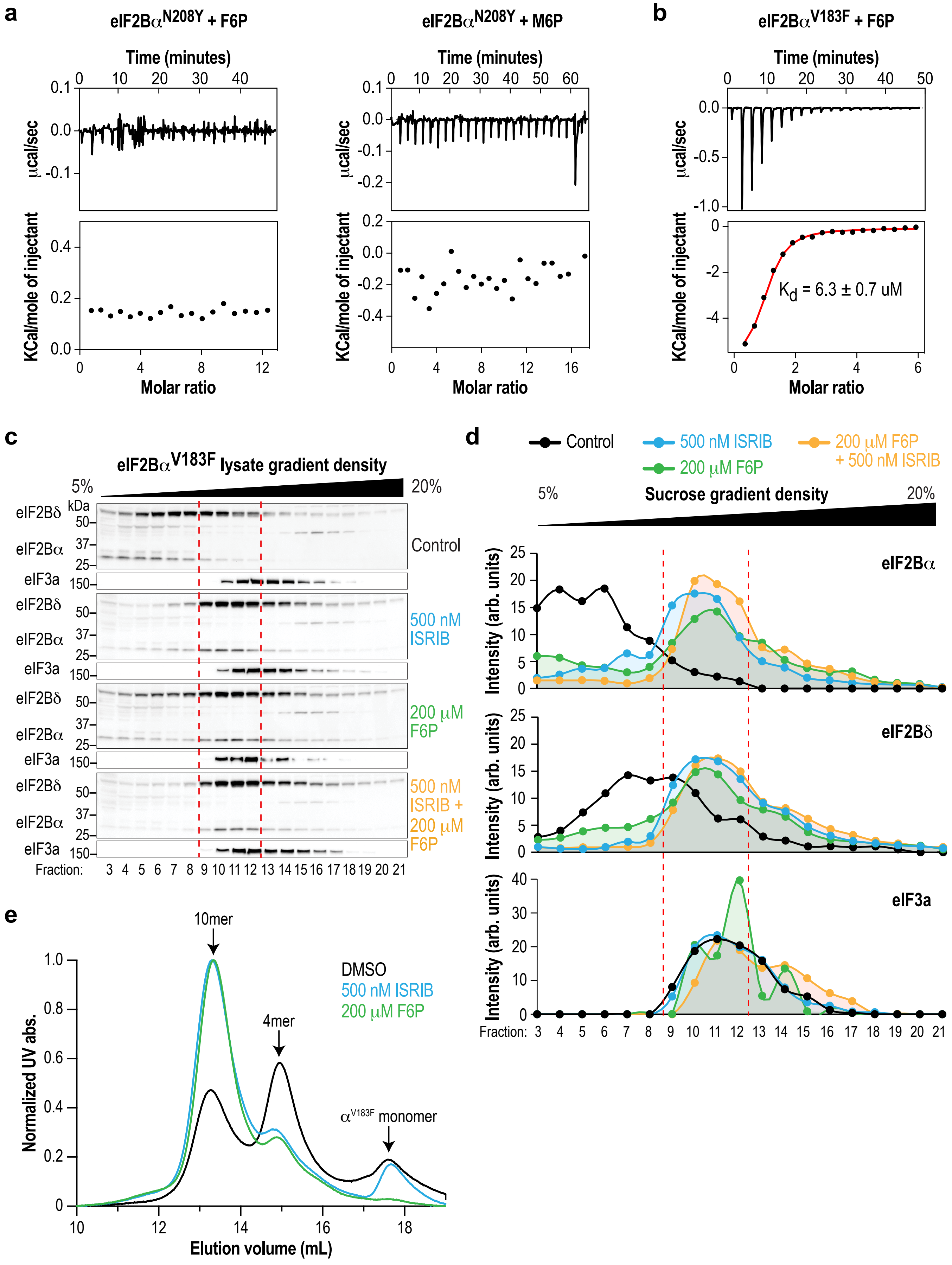

Supplementary Figure 11. **Sugar phosphate binding is abolished by the eIF2Bα^{N208Y} mutant but maintained in the eIF2Bα^{V183F} mutant. a** eIF2Bα^{N208Y} does not interact with F6P or M6P. **b** eIF2Bα^{V183F} interacts with F6P similar to wild-type eIF2Bα. In **a** and **b**, the upper subpanel shows the baseline-subtracted thermogram. The bottom subpanel represents the binding isotherm, with the red line indicating the fit curve, if available. **c** Sucrose gradient centrifugation of eIF2Bα^{V183F} HEK293T lysate treated with ISRIB (blue), F6P (green) or both (orange). Fractions from the sucrose gradient were subjected to SDS-PAGE followed by immunoblotting with the indicated antibodies. eIF3a was used as an internal control. **d** Quantification of **c** normalized by total intensity of each subunit in its respective gradient. Data shown are representative of 3 replicates of each experiment. In **c** and **d**, dashed red lines demark the boundary of the decameric eIF2B peak. **e** Size-exclusion chromatograms of purified recombinant eIF2B(βδγε) + eIF2Bα^{V183F} in the absence or presence of ISRIB or F6P. The elution positions of the $(\alpha\beta\delta\gamma\varepsilon)_2$ decamer, (βδγε) tetramer and α^{V183F} monomer are indicated.

Supplementary Table 1. **Cryo-EM data collection, refinement and validation statistics**

	eIF2B-F6P (EMD-22924) (PDB 7KMF)
**Data collection and processing**	
Magnification	130,000
Voltage (kV)	300
Electron exposure (e–/Å^2)	44.33
Defocus range (μm)	-1.0 to -2.4
Pixel size (Å)	1.04
Symmetry imposed	C2
Initial particle images (no.)	736,044
Final particle images (no.)	73,704
FSC threshold	0.143
Map resolution range (Å)	2.9 – 6.0
**Refinement**	
Initial model used (PDB code)	6CAJ
Model Resolution (Å)	3.0
FSC threshold	0.143
Model resolution range (Å)	2.9
Map sharpening *B* factor (Å^2)	-106.6
Model Composition	
Non-hydrogen atoms	22875
Protein residues	3005
Ligands	2
B factors (Å^2)	
Protein	104.02
Ligand	82.94
R.m.s. deviations	
Bond lengths (Å)	0.009
Bond angles (°)	1.066
Validation	
MolProbity score	1.88
Clashscore	7.69
Poor rotamers (%)	0.21
Ramachandran plot	
Favored (%)	92.76
Allowed (%)	7.24
Disallowed (%)	0

Supplementary Table 2. **X-ray data collection and refinement statistics (molecular replacement)**

	eIF2Bα-M6P (PDB 7KMA)
**Data collection**	
Space group	P 1 21 1
Cell dimensions	
a, *b*, *c* (Å)	71.2, 155.5, 140.1
α, β, γ (°)	90, 103.89, 90
Resolution (Å)	55.61 – 2.7 (2.797 – 2.70)
R_{sym} or R_{merge}	0.0364 (0.3218)
$I / \sigma I$	13.35 (2.51)
Completeness (%)	93.72 (99.64)
Redundancy	1.9 (1.9)
**Refinement**	
Resolution (Å)	2.7
No. reflections	76117 (8075)
R_{work} / R_{free}	0.1907/0.2381
No. atoms	
Protein	16887
Ligand/ion	132
Water	153
B-factors	
Protein	68.36
Ligand/ion	55.65
Water	55.45
R.m.s. deviations	
Bond lengths (Å)	0.008
Bond angles (°)	0.93

*Values in parentheses are for highest-resolution shell.
