# Supplementary material for: Sugar phosphate activation of the stress sensor eIF2B
Source: Nat Commun. 2021 Jun 8;12:3440. doi: 10.1038/s41467-021-23836-z (PMC8187479; doi:10.1038/s41467-021-23836-z)
Supplement: Supplementary file 4 — Description of additional supplementary files [file 41467_2021_23836_MOESM4_ESM.docx]

Description of additional supplementary information

Title: Supplementary Data 1

Description: Results of MIDAS analysis for metabolite binding to eIF2B α

Title: Supplementary Data 2

Description: Results of GEF assay for metabolite enhancement/inhibition of eIF2B activity
